# Superhydrated Zwitterionic Hydrogel with Dedicated Water Channels Enables Nonfouling Solar Desalination

**DOI:** 10.1007/s40820-025-01937-4

**Published:** 2025-12-08

**Authors:** Panpan Zhang, Hanxue Liang, Yawei Du, Haiyang Wang, Yaqi Tian, Jingtao Bi, Lei Wang, Zhiyuan Guo, Jing Wang, Zhi-Yong Ji, Liangti Qu

**Affiliations:** 1 Engineering Research Center of Seawater Utilization Technology of Ministry of Education, Hebei Collaborative Innovation Center of Modern Marine Chemical Technology, Tianjin Key Laboratory of Chemical Process Safety, School of Chemical Engineering and Technology, Hebei University of Technology, Tianjin, 300130 People’s Republic of China; 2 Research Center for Analytical Sciences, Tianjin Key Laboratory of Biosensing and Molecular Recognition, College of Chemistry, Nankai University, Tianjin, 300071 People’s Republic of China; 3 Key Laboratory of Organic Optoelectronics & Molecular Engineering, Ministry of Education, Department of Chemistry, Tsinghua University, Beijing, 100084 People’s Republic of China

**Keywords:** Zwitterionic hydrogel, Strong hydration, Nonfouling ability, Sustainable solar desalination, Complex marine environments

## Abstract

Solar-driven interfacial desalination (SID) offers a sustainable route for freshwater production, yet its long-term performance is compromised by salt crystallization and microbial fouling under complex marine conditions. Zwitterionic polymers offer promising nonfouling capabilities, but current zwitterionic hydrogel-based solar evaporators (HSEs) suffer from inadequate hydration and salt vulnerability. Inspired by the natural marine environmental adaptive characteristics of saltwater fish, we report a superhydrated zwitterionic poly(trimethylamine N-oxide, PTMAO)/polyacrylamide (PAAm)/polypyrrole (PPy) hydrogel (PTAP) with dedicated water channels for efficient, durable, and nonfouling SID. The directly linked N⁺ and O⁻ groups in PTMAO establish a robust hydration shell that facilitates rapid water transport while resisting salt and microbial adhesion. Integrated PAAm and PPy networks enhance mechanical strength and photothermal conversion. PTAP achieves a high evaporation rate of 2.35 kg m^−2^ h^−1^ under 1 kW m^–2^ in 10 wt% NaCl solution, maintaining stable operation over 100 h without salt accumulation. Furthermore, PTAP effectively resists various foulants including proteins, bacterial, and algal adhesion. Molecular dynamics simulations reveal that the exceptional hydration capacity supports its nonfouling properties. This work advances the development of nonfouling HSEs for sustainable solar desalination in real-world marine environments.

**Supplementary Information:**

The online version contains supplementary material available at 10.1007/s40820-025-01937-4.

## Highlights

- A superhydrated zwitterionic poly(trimethylamine N-oxide, PTMAO)/polyacrylamide/polypyrrole hydrogel (PTAP) with dedicated water channels is proposed for nonfouling solar desalination.
- The directly linked N⁺ and O⁻ groups in PTMAO establish a robust hydration shell that facilitates rapid water transport while resisting salt and microbial adhesion.
- PTAP achieves a high evaporation rate of 2.35 kg m^−2^ h^−1^ under 1 kW m^–2^ in 10 wt% NaCl solution for 100 h without salt accumulation, and can resists various foulants including proteins, bacterial, and algal adhesion.

**Supplementary Information:**

The online version contains supplementary material available at 10.1007/s40820-025-01937-4.

## Graphical abstract

**Figure:**
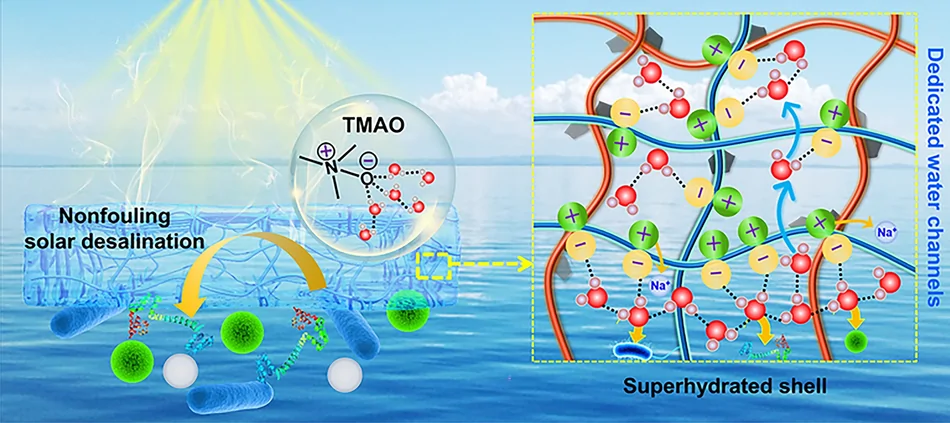

## Introduction

Global water resources are becoming increasingly scarce, posing a critical challenge to sustainable development and human well-being worldwide [1–3]. Solar-driven interfacial desalination (SID) offers a promising route for sustainable freshwater production by harnessing abundant solar energy [4–6]. Common materials for solar evaporators primarily include carbon materials and hydrogels [7–9]. Among them, hydrogel-based solar evaporators (HSEs), fabricated from materials such as polyvinyl alcohol (PVA) [10–12], chitosan (CS) [13–15], and sodium alginate (SA) [16], are extensively employed in SID system due to their advantages of affordability, facile assembly, excellent biocompatibility, and reduced enthalpy of water evaporation. However, the complex marine environments contain various interfering pollutants, including inorganic salts, and microorganisms such as proteins, bacteria, and algae [17–19]. Along with continuous evaporation of water, non-volatile salts and microorganisms are prone to accumulate within the porous structure or at the photothermal interface of HSEs, ultimately compromising their long-term performance and operational stability [20].

Currently, various HSEs achieve ongoing SID with outstanding salt-resistant capacities, facilitated by ion diffusion equilibrium [21–23], Donna effect [24–26], and polyelectrolyte effect [27]. However, microbial accumulation on the surface of HSEs can lead to material degradation, resulting in structural failure and a decline in SID performance [28]. Additionally, the nutrients that accumulate on the surface provide a favorable environment for microbial attachment and proliferation, which causes irreversible marine biofouling and weakens the long-term durability [29]. Hence, it is imperative to develop HSEs that excel in both water evaporation performance and nonfouling properties, particularly in harsh marine environments, to ensure sustainable SID.

Zwitterionic polymers, which contain both positively charged and negatively charged groups with the overall net charge of zero, have emerged as promising candidates for nonfouling purposes [30]. Till now, mainly two types of zwitterionic polymers including poly(sulfobetaine methacrylate) (PSBMA) and poly(3-(1-(4-vinylbenzyl)-1Himidazol-3-ium-3-yl) propane-1-sulfonate) (PVBIPS) have been proposed as HSEs for efficient high-salinity SID ascribed to their anti-polyelectrolyte effect [31–33]. That is, the anti-polyelectrolyte effect of zwitterionic polymers is able to enhance the hydration of polymeric networks for rapid water uptake and improved salt tolerance. The hydration capacity along with the nonfouling property of zwitterionic polymers improves as the intramolecular distance between the positively and negatively charged sites decreases. Nevertheless, currently available PSBMA and PVBIPS exhibit relatively longer intramolecular distances between the positively and negatively charged groups, resulting in insufficient surface hydration to provide the desired nonfouling ability. Simultaneously, the surface hydration of these zwitterionic HSEs is dramatically disrupted by salt ions, which compromises their salt resistance. Furthermore, the nonfouling ability of these zwitterionic HSEs, especially in the harsh marine environments, encompassing various proteins, bacteria, and algae, has not been systematically investigated. Consequently, it remains challenging to develop effective zwitterionic HSEs with superhydrated polymeric networks that enable highly-efficient, sustainable and nonfouling SID in such environments.

**Fig. 1:**
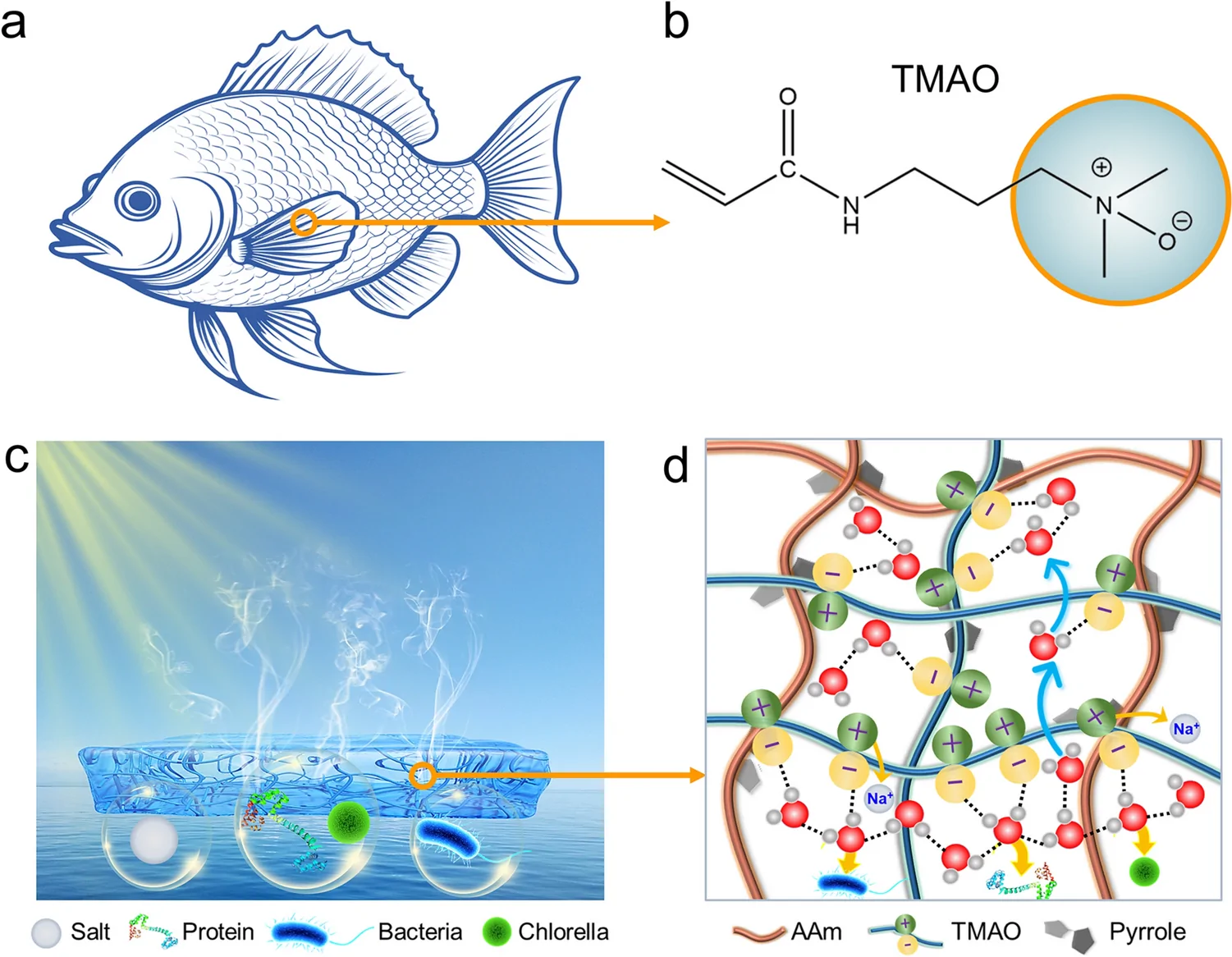
**a, b** Zwitterionic TMAO inspired by natural marine environmental adaptive characteristics of saltwater fish. **c, d** Nonfouling SID design of superhydrated PTAP with dedicated water channels

Herein, inspired by the natural marine environmental adaptive characteristics of saltwater fish, we propose a superhydrated zwitterionic poly(trimethylamine N-oxide) (PTMAO)/polyacrylamide/polypyrrole hydrogel (PTAP) with dedicated water channels for nonfouling SID (Fig. 1). Among them, the zwitterionic PTMAO carries directly linked positively charged N^+^ and negatively charged O^−^ groups. The O^−^ can form strong bonding interactions with water molecules, allowing them transport to the photothermal interface for fast evaporation. Simultaneously, the N^+^ can strongly repel salt ions and prevents damage to the superhydrated shell, ensuring exceptional nonfouling performance in continuous SID process. The incorporation of polyacrylamide (PAAm) and polypyrrole (PPy) enhances the mechanical properties and light absorption ability of PTAP, thereby improving the suitability, durability, and energy utilization efficiency. As such, PTAP exhibits an excellent water evaporation rate of 2.35 kg m^−2^ h^−1^ (1 kW m^−2^) in a high salinity of 10 wt% NaCl solution, and there is no significant salt accumulation on PTAP surface over 100 h of prolonged use. Additionally, PTAP is able to effective against the attachment of foulants including proteins, bacteria, and algae. Molecular dynamics (MD) simulations also verify the nonfouling mechanism via extraordinary hydration of PTAP at molecular level. This well-designed PTAP enriches the arsenal of nonfouling HSEs for sustainable SID even in the harsh marine environments and is expected to benefit a wide range of applications.

## Experimental Section

### Synthesis of TMAO Monomer

The zwitterionic monomer TMAO was synthesized via a one-step oxidation process. Briefly, 200 mg of diethylenetriaminepentaacetic acid (DTPA) was fully dissolved in 15 mL of deionized water, followed by the slow addition of 10 mL of 30% hydrogen peroxide (H_2_O_2_) solution. The mixture was subsequently heated to 60 °C and transferred into a three-necked flask pre-filled with oxygen (O_2_). Separately, 3.6 g of *N*,*N*-dimethylaminopropyl acrylamide was completely dissolved in 10 mL of deionized water and added dropwise into the flask. The reaction was maintained at 60 °C under continuous stirring for 6 h. Upon completion, the mixture was cooled to room temperature, and the resulting product was extracted multiple times with dichloromethane. The final product was obtained as a colorless, viscous TMAO monomer by freeze-drying. The yield of TMAO synthesis is usually high, reaching up to 85%. PTAP can be prepared on a large scale.

### Preparation of PTAPs

Various amounts of TMAO (100, 200, 300, and 400 mg), AAm (560 mg), the cross-linker MBAA, and the photoinitiator 2959 were dissolved in 4 mL of deionized water. The concentrations of MBAA and 2959 were set at 1.5 and 0.15 wt% relative to the mass of TMAO, respectively. The mixture was homogenized by ultrasonication and subsequently photopolymerized under UV irradiation for 30 min to form PTMAO/PAAm hydrogels. The as-prepared hydrogels were immersed in deionized water overnight to achieve full swelling, followed by transfer into 10 mL of deionized water containing 69 μL of pyrrole and 1 mL of phytic acid for 24 h. In situ polymerization was then conducted in a 0.1 mol L^−1^ ammonium persulfate solution for 1 h. Residual pyrrole monomers were removed by extensive washing with deionized water, yielding PTMAO/PAAm/PPy hydrogels (denoted as PTAPs). The hydrogels were labeled PTAP1, PTAP2, PTAP3, and PTAP4 corresponding to TMAO contents of 100, 200, 300, and 400 mg, respectively.

### SID Experiments

The SID device was designed with a three-layer configuration including a solar evaporator (20 mm × 20 mm active area), a polystyrene foam (PS) thermal insulation layer (10 mm thickness), and hydrophilic cotton rods embedded within the PS matrix to serve as water transport channels. The assembled device was submerged in a water reservoir, with the cotton rods ensuring a continuous water supply to the photothermal layer. The SID performance was evaluated under sunlight irradiation (1 kW m^−2^). Mass loss was monitored hourly using an analytical balance with a resolution of 0.1 mg to calculate the evaporation rate.

### Anti-Protein Measurements

A standard calibration curve for the quantification of bovine serum albumin (BSA) adsorption was constructed by measuring the UV–vis absorbance at 280 nm for BSA solutions at varying concentrations. Hydrogel samples were first equilibrated in deionized water for 24 h, followed by immersion in a 0.5 mg mL^–1^ BSA solution for another 24 h. Subsequently, the samples were carefully extracted with tweezers and gently rinsed with phosphate-buffered saline (PBS). PBS is employed to remove loosely bound or nonspecifically adsorbed BSA, thereby allowing accurate assessment of the hydrogel’s true nonfouling capability based on firmly attached proteins. This is a standard protocol in nonfouling studies to distinguish between reversible and irreversible adsorption [34, 35]. They were then washed thoroughly with ultrapure water to remove strongly bound BSA. The pH is 7.4, and the ionic strength is ~ 10 mM (standard PBS). The absorbance of the eluates was recorded at λ = 280 nm using UV–vis spectroscopy and compared to the pre-established calibration curve to determine surface-adsorbed BSA content.

### Anti-Bacterial Experiments

Bacterial adhesion experiments were conducted with *S. aureus* and *E. coli* using primary cultures grown in lysogeny broth (LB) and incubated at 37 °C until reaching the exponential growth phase. Under a laminar flow hood, 0.1 mL of the bacterial culture was diluted with a 0.9% sterile NaCl solution to the desired concentrations, with the same saline solution used as a blank control. Absorbance at λ = 600 nm was measured to adjust the bacterial suspensions to an optical density (OD_600 nm_ = 0.6–0.7). Sterilized hydrogel samples were immersed in these bacterial suspensions and incubated at 4 °C for 24 h. After incubation, the optical density of the remaining bacterial suspension was assessed to determine the hydrogel’s anti-adhesion effectiveness. To prepare for antimicrobial experiments, the bacterial suspension was serially diluted with sterile medium to an optimal concentration (OD_600 nm_ = 0.20). Following standardized protocols, the diluted suspension was aseptically plated onto nutrient agar using a spiral plater and incubated at 37 °C for 24 h under aerobic conditions. The number of bacterial colonies on the agar plates was then counted to evaluate the antimicrobial properties of the hydrogel samples.

### Algae Resistance Evaluation

A hydrogel sample (10 mm × 10 mm) was cut from the fully swollen and saturated hydrogel. The hydrogel was then immersed in a 2 mg mL^–1^ Chlorella, Diatoms and Chrysophytes solutions and co-cultured for 24 h. After incubation, the sample was carefully retrieved using tweezers and rinsed three times with deionized water. The distribution density of algae on the hydrogel surface was observed under an inverted biological microscope.

## Results and Discussion

### Fabrication and Characterization

**Fig. 2:**
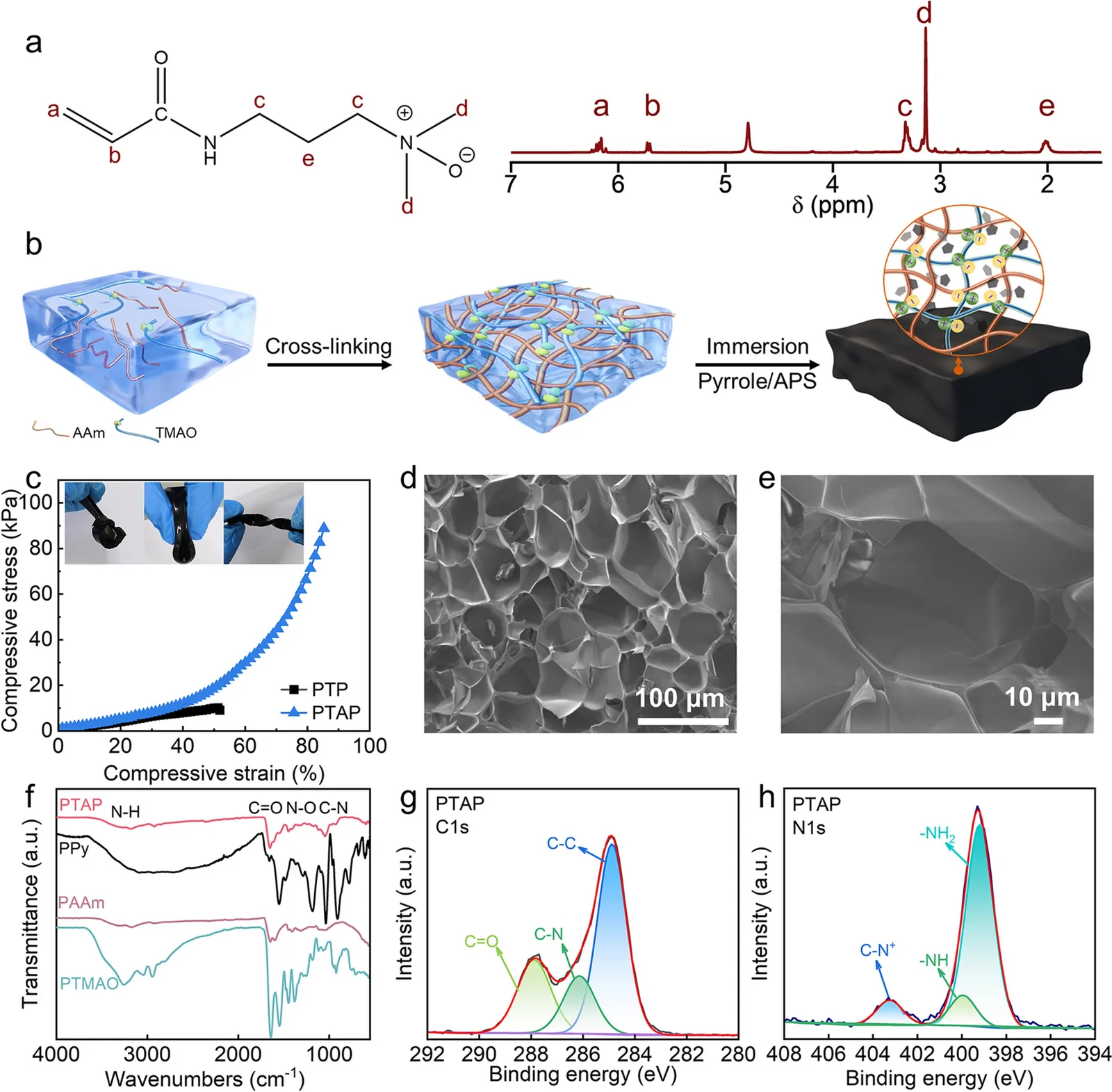
**a** Molecular formula and ^1^H-NMR spectra of TMAO monomer. **b** Schematic diagram of the preparation process of PTAP. **c** Stress–strain curves of PTP and PTAP under uniaxial compression after immersion in seawater. Inset photographs are PTAPs undergoing rotating, folding, and twisting. **d, e** SEM images of PTAP at different magnifications, showing homogeneous porous structure. **f** FTIR spectra of PTMAO, PAAm, PPy, and PTAP. High-resolution **g** C 1*s*, and **h** N 1*s* XPS spectra of PTAP, respectively

The TMAO monomer is synthesized by oxidizing *N,N*-dimethylaminopropyl acrylamide (DMAPA) using a stronger oxidizing agent (50% hydrogen peroxide, H_2_O_2_) to ensure a high conversion of DMAPA [36]. The proton nuclear magnetic resonance (1H-NMR) spectra verify that characteristic peaks can be well recognized and assigned to each proton in the TMAO molecule, proving the successful synthesis of the target TMAO monomer (Figs. 2a, S1 and S2). Typically, the dual-network PTMAO/PAAm hydrogel is prepared by polymerization and gelation of TMAO and AAm monomers, where *N,N*’-methylenebisacrylamide (MBAA) and 2-hydroxy-2-methylpropiophenone act as cross-linker and UV initiator, respectively. Subsequently, the transparent PTMAO/PAAm hydrogel is immersed in a pyrrole solution to allow the penetration of pyrrole monomer into the hydrogel skeleton, followed by in situ polymerization in the presence of an ammonium persulfate (APS) initiator to form polypyrrole-loaded photothermal PTAP (Figs. 2b and S3). Pure zwitterionic PTMAO hydrogel tends to have poor mechanical properties due to its high water content, low polymer chain density and weak contacts, limiting their suitability and durability for practical SID applications. Accordingly, a strong interpenetrating chain entanglement of PAAm through chemical cross-linking could form in the PTMAO hydrogel, which can effectively enhance the mechanical properties of PTAP in saline environments (Fig. S4). The superior mechanical performance of PAAm arises from its flexible carbon backbone, the abundance of amide groups capable of forming hydrogen bonds, and its excellent compatibility for cross-linking and hybridization. These characteristics make PAAm widely utilized as a structural enhancer and toughening agent in hydrogel [37, 38]. PTAP can be prepared over a large area and remains structurally intact after rotating, folding and twisting (Fig. S5). PTAP displays superior compressive stress of 88.8 kPa in seawater environments compared to PTMAO/PPy hydrogel (PTP, 10.1 kPa) without the introduction of PAAm polymeric network (Fig. 2c). Scanning electron microscopy (SEM) images of PTAP reveal homogeneous porous structure, which is beneficial for light trapping and water transport (Fig. 2d, e). Energy-dispersive X-ray spectroscopy (EDS) confirm the presence of C, N, and O as the primary elements within the PTAP. Corresponding elemental mapping images reveal their uniform distribution throughout the microstructure of PTAP (Fig. S6). Fourier transform infrared spectroscopy (FTIR) stretching vibration of N–H, and a peak at 1650 cm^−1^ associated with the stretching vibration of C=O. Characteristic peaks observed at 1250 and 1050 cm^−1^ are assigned to the stretching vibration of N–O and C–N, respectively (Fig. 2f) [37, 39]. These features closely match those of PTMAO, confirming its successful incorporation (Fig. S1b). The high-resolution C 1*s* X-ray photoelectron spectroscopy (XPS) of PTAP reveal three typical carbon bonds of C − C (284.7 eV), C=O (287.9 eV), and C–N (286.0 eV) (Fig. 2g) [40]. The N 1*s* XPS spectra of PTAP confirm the apparent peaks at 399.8 and 399.2 eV attributed to –NH and –NH_2_, and a peak at 402.8 eV associated with –C–N^+^ that originates from TMAO (Figs. 2h and S7) [41]. These chemical characterizations validate the presence of zwitterionic PTMAO with directly linked positively charged N^+^ and negatively charged O^−^ groups in the PTAP.

### Solar-Driven Water Evaporation Performance

**Fig. 3:**
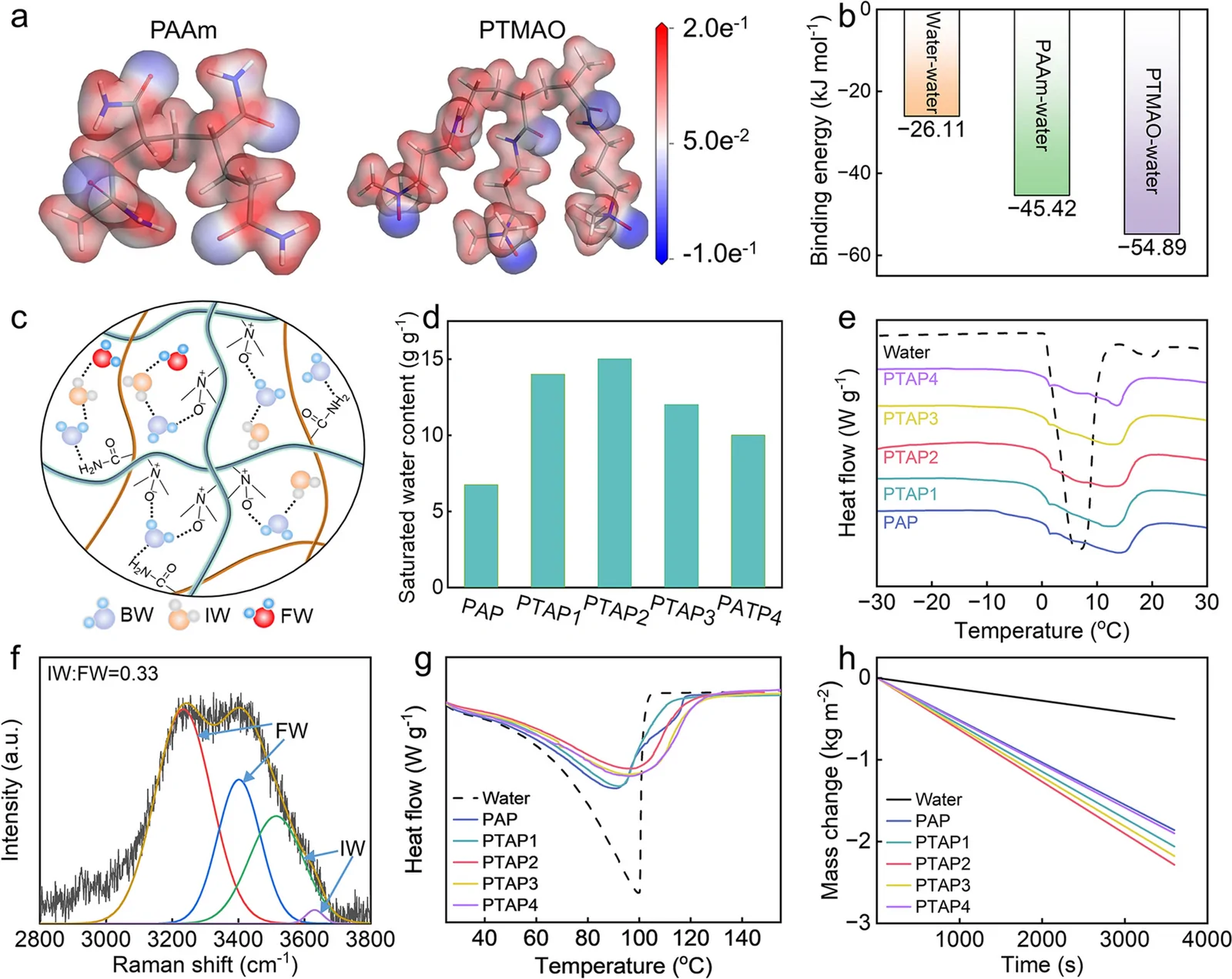
**a** Electrostatic potential mapping of PTMAO molecules. **b** Binding energy of water–water, PAAm–water, and PTMAO–water molecules according to the DFT calculations. **c** Schematic diagram of water states within PTAPs. **d** Saturated water content of PAP and PTAPs. **e** DSC curves of melting behavior composed of water and water frozen in PAP and PTAPs. **f** Raman spectra with fitting curves showing FW and IW in PTAP2. **g** DSC thermograms of bulk water and water in PAP and PTAPs. **h** Mass change of water, PAP and PTAPs over time under 1 kW m^–2^

Zwitterionic PTMAO that are entirely superhydrophilic are able to from very strong interactions with water, and thus regulating water states including bound water (BW), intermediate water (IW), and free water (FW) in PTAP [42, 43]. The density functional theory (DFT) calculations reveal that the binding energy for PTMAO–water interactions (–54.89 kJ mol^−1^) is much higher compared to water–water (–26.11 kJ mol^−1^) and PAAm–water (– 45.42 kJ mol^−1^) interactions (Fig. 3a, b) [37]. As such, by adjusting the PTMAO content in PTAP, the water states can be optimized to increase the IW content, reduce the energy required for evaporation, and enhance the water evaporation rate (Fig. 3c) [44, 45]. It is important to note that the mechanical properties of pure PTMAO hydrogel are poor, and further increases in PTMAO content gradually weaken the mechanical properties of PTAP. Therefore, the PTMAO content must be optimized to balance PTAP’s mechanical properties and solar-driven water evaporation performance. To achieve this, PTAPs with varying PTMAO contents, 100, 200, 300, and 400 mg of PTMAO per 560 mg of AAm, were prepared and designated as PTAP1, PTAP2, PTAP3, and PTAP4, respectively (Figs. S8 and S9). All PTAPs display superior compressive stress in seawater environments compared to pure PTMAO and PTMAO/PPy hydrogel (PTP) without the introduction of PAAm polymeric network (Fig. S10). However, as the PTMAO content increases, the mechanical properties of PTAPs tend to deteriorate. The saturated water contents of PTAPs initially increase and then decrease, but overall remain higher than that of PAP.

PTAP2 delivers a highest saturated water content of 15.0 g g^−1^ (Fig. 3d). The introduction of superhydrophilic PTMAO enables strong hydrogen bonding with water molecules, regulating the water states within PTAPs. From PAP to PTAP2, the IW content in these hydrogels gradually increase as the PTMAO content rise up. However, for PTAP3 and PTAP4, with further increases in PTMAO content, the degree of cross-linking is high and more water molecules are BW, leading to a reduction in saturated water content. Differential scanning calorimetry (DSC) ice melt characterization confirms a decreasing trend in melt enthalpy from PAP to PTAP4, attributed to the increase in BW content, as BW does not produce an endothermic peak in the DSC ice melt test (Fig. 3e) [46, 47]. Raman spectra with fitting curves verify that PTAP2 owns the highest IW/BW ratio (Figs. 3f and S11) [48, 49]. As a result, PTAP2 has the lowest energy consumption with an evaporation enthalpy of 1450 J g^−1^ (Fig. 3g). Benefiting from the incorporation of PPy, a superior sunlight-absorbing material, PAP and PTAPs exhibit ultra-high light absorption > 98% across the entire solar spectrum (250 − 2500 nm) (Fig. S12a). Under 1 kW m^–2^ solar irradiation, the surface temperatures of PAP and PTAPs are significantly higher than that of pure water (Fig. S12b). Notably, PTAP2 delivers the highest water evaporation rate, reaching 2.28 kg m^−2^ h^−1^, with an energy utilization efficiency of 91.8%, outperforming pure water, PAP, and other PTAPs (Figs. 3h and S13).

### Salt-Resistant Ability and Its Mechanism Exploration at Molecular Level

**Fig. 4:**
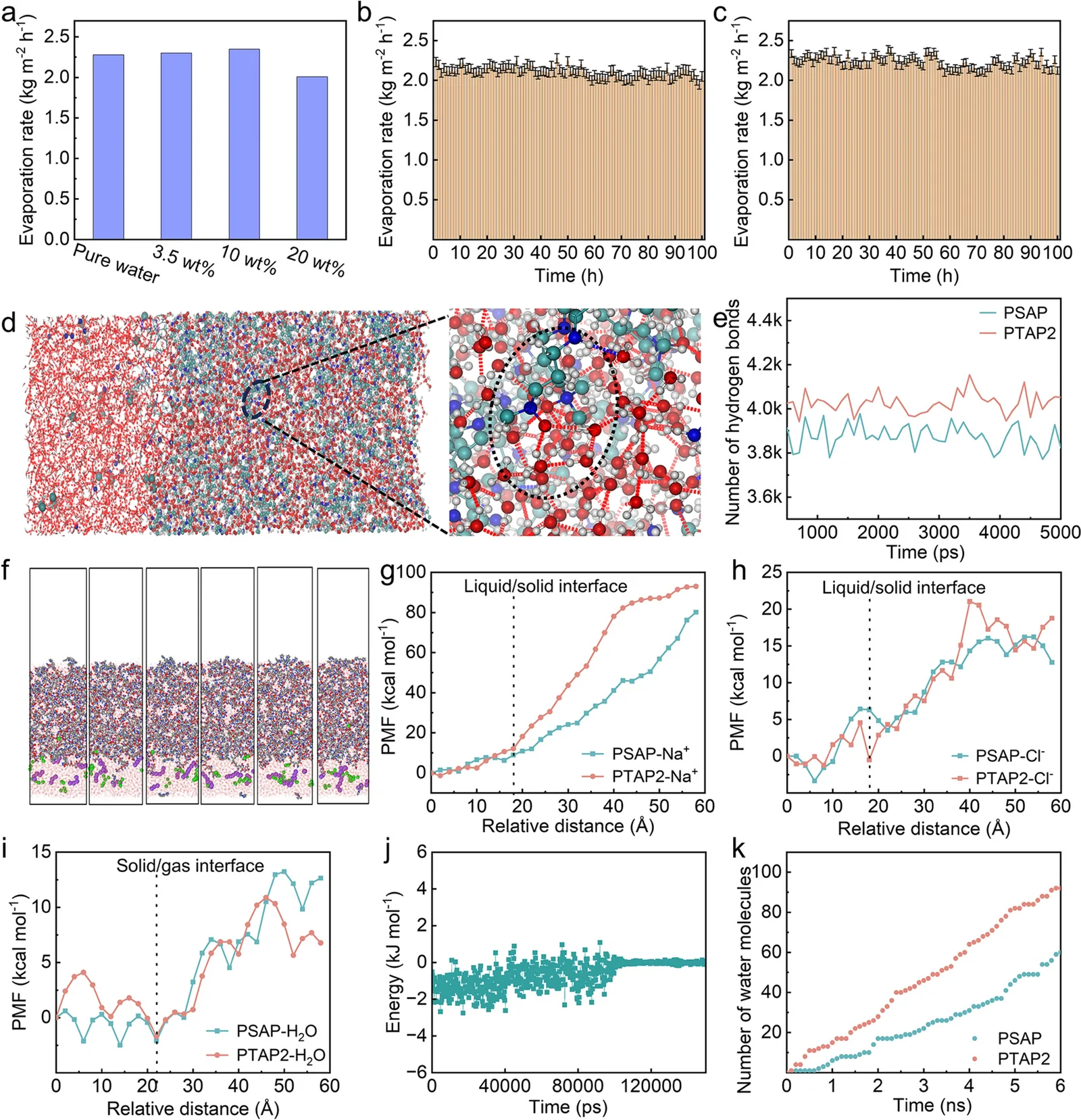
**a** Evaporation rates of PTAP2 in pure water and different brines. **b, c** Long-term SID test of PTAP2 over 100 h (8 h per day) in 3.5 wt% and 10 wt% brines, respectively. **d** Schematic diagram of simulated water molecule transport in PTAP2 in 3.5 wt% NaCl solution. The enlarged image shows the strong hydrogen bonding of PTMAO with surrounding water molecules in PTAP2. **e** Number of hydrogen bonds of PSAP and PTAP2 using the last 5000 ps of the simulation trajectory. **f** Simulated snapshots of the salt ions states during evaporation in PTAP2. PMF curves of **g** PSAP-Na^+^ and PTAP2-Na^+^, **h** PSAP-Cl^−^ and PTAP2-Cl^−^ at the liquid/solid interface, and **i** PSAP-H_2_O and PTAP2-H_2_O at the solid/gas interface along the direction of evaporation, respectively. **j** At the solid/gas interface, the energy required for water vapor escape from PTAP2. **k** Number of water molecules evaporated through the solid/gas interface of PSAP and SBMA in 3.5 wt% NaCl solution

The long-term stability of water evaporation performance using PTAP2 were systematically evaluated in brines with varying salinities (3.5 wt%, 10 wt%, and 20 wt%) to demonstrate its salt resistance. PTAP2 delivers the water evaporation rate of 2.30, 2.35, and 2.01 kg m^−2^ h^−1^ in 3.5, 10, and 20 wt% brines under the solar irradiation of 1 kW m^−2^ (Figs. 4a and S14). It is noteworthy that with increasing salt concentration, the water evaporation rate of PTAP2 initially increases and then decreases, reaching its maximum value in 10 wt% brine. This phenomenon may be attributed to the ionic species screening effects in the saline solution at higher concentrations, which neutralizes the positive and negative charges on the zwitterionic polymer. As a result, the electrostatic interactions between zwitterionic polymers are weakened, leading to a more relaxed hydrogel network that facilitates water transport to the evaporation interface (Fig. S15) [31]. Subsequently, we evaluated the long-term salt resistance of PTAP2. In an initial 3.5 wt% brine (100 mL), no salt crystallization is observed on the surface of PTAP2 until the brine is completely evaporated (Fig. S16). Even under continuous operation for 8 h in highly concentrated brine (20 wt%), PTAP2 exhibits no significant salt accumulation on its evaporation interface, and the water evaporation rate remains stable. In contrast, under the same conditions, the evaporation interface of PAP is almost entirely covered with solid salt, and its evaporation rate decreases by 22.5% (Fig. S17). These phenomena indicate that the introduction of PTMAO endows PTAP2 with excellent salt resistance. Furthermore, PTAP2 was subjected to extended continuous testing in 3.5 and 10 wt% brines (100 h total, in 8 h intervals). The declines in water evaporation rates are minimal, with average values maintain at 2.15 and 2.23 kg m^−2^ h^−1^ (1 kW m^−2^), respectively, well confirming the outstanding long-term salt resistance of PTAP2 (Fig. 4b, c). During the continuous operation in both 3.5 wt% and 10 wt% brines, no visible swelling, deformation, or structural collapse was observed over the testing period (Fig. S18). FTIR of PTAP2 before and after use reveals no observable shifts in the characteristic peaks of the major functional groups, confirming the PTAP2’s excellent chemical stability throughout the long-time SID process (Fig. S19). Notably, the designed PTAP2 exhibits a clear advantage in evaporation rate under both low- and high-salinity conditions compared with previously reported solar evaporators (Table S1) [50, 51].

In PTAP2, the zwitterionic PTMAO features closely positioned positively charged N⁺ and negatively charged O^−^. The O^−^ engages in strong hydrogen bonding with water molecules, enhancing their transport to the photothermal interface and thereby accelerating evaporation. Simultaneously, the N^+^ effectively repels salt ions, preserving the superhydrated shell and ensuring excellent nonfouling performance during continuous SID operation. Accordingly, the strong hydration-induced salt-resistant mechanism of PTAP2 at molecular level was explored using MD simulations. Firstly, we performed MD simulations of a TMAO small molecule in a saline solution [52]. The N^+^ in TMAO is shielded by three methyl groups and does not interact with surrounding water molecules. Therefore, the water molecules directly associated with the oxygen atom is primarily responsible for TMAO’s strong hydration. Approximately, three hydrogen bonds are formed between water molecules and the oxygen atom of TMAO, contributing to the formation of a stable superhydrated shell (Fig. S20a). Radial distribution function g(*r*) displays a distinct peak between the oxygen atom of TMAO and the hydrogen atoms of surrounding water molecules, situated within 1.0–1.7 Å, confirming the presence of strong hydrogen bonding (Fig. S20b). The close spatial arrangement of O^−^ and N^+^ in TMAO creates electrostatic repulsion that prevents Na^+^ from binding tightly to O^−^. This combined spatial and electrostatic effect means that the superhydrated shell of TMAO acts as a barrier to Na^+^ penetration, and thus superhydrated shell is little altered by the addition of NaCl (Fig. S21).

Subsequently, the simulation models based on PTAP2 and PSAP (zwitterionic PSBMA/polyacrylamide/polypyrrole hydrogel) were built to analyze the water evaporation behavior and strong hydration-induced salt-resistant mechanism (Figs. S22–S27). Since PSBMA is a commercially available zwitterionic polymer, it is commonly used in the construction of solar evaporators for SID. Therefore, we replaced PTMAO with PSBMA to prepare PSAP as a control sample, aiming to investigate the anti-salt mechanism of PTAP2. We simulated the evaporation of water molecules across the PSBMA and PTAP2 in 3.5 wt% NaCl solution using MD simulations based on the large-scale atomic molecular parallel simulator (LAMMPS) [53, 54]. The MD simulation results validate the stronger hydration of PTAP2 compared with PSAP (Figs. 4d and S28). Meanwhile, the number of hydrogen bonds in PTAP2 are greater than that of PSAP (Figs. 4e and S29). As water molecules evaporate, no substantial salt ion infiltration is detected within PSBMA or PTAP2 (Figs. 4f and S30). At the liquid/solid interface, the potential of mean force (PMF) for PTAP2-Na⁺ and PTAP2-Cl⁻ is notably higher than that for PSAP-Na⁺ and PSAP-Cl⁻, suggesting a greater transmembrane energy barrier and enhanced salt resistance in PTAP2 (Figs. 4g, h and S31). In contrast, at the solid/gas interface, the energy required for water escape from PTAP2 is markedly lower than that from PSAP, thereby promoting more efficient water evaporation at the PTAP2 evaporation interface (Figs. 4i–k and S32–S34). Similarly, experimental data based on PSAP and PTAP2 further confirm that PTAP2 exhibits superior water evaporation performance and enhanced salt resistance compared to PSAP (Fig. S35). These findings indicate that the directly connected positively charged N⁺ and negatively charged O^−^ within the PTMAO of PTAP2 contribute to its strong hydration capability, which indicates its outstanding salt resistance and strengthens its stability and durability in long-term SID applications.

### Anti-Biofouling Performance

**Fig. 5:**
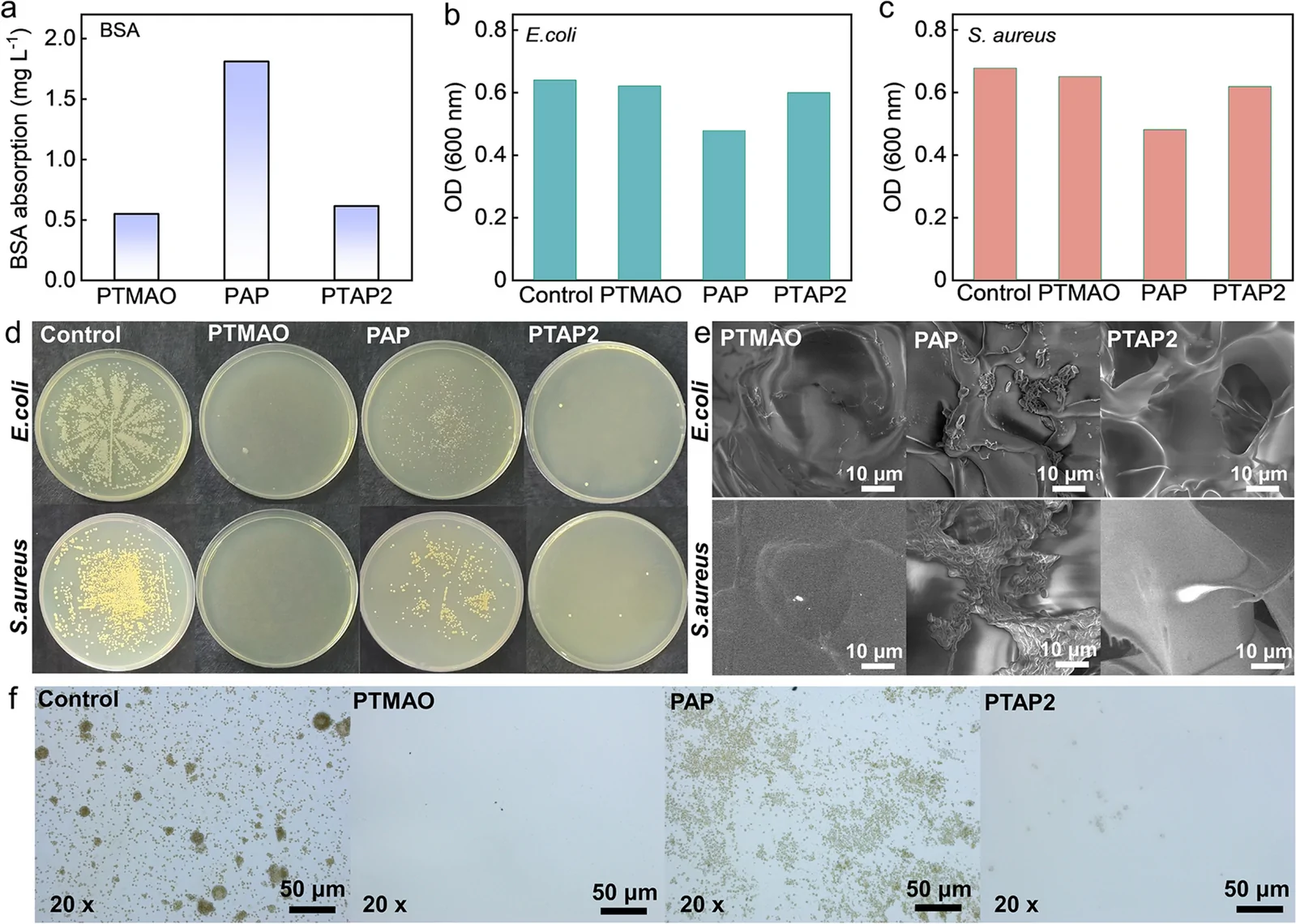
**a** Adsorption amount of BSA on the surfaces of blank control, PTMAO, PAP, and PTAP2. The OD_600nm_ values of bacterial suspensions including **b** *E. coli* and **c** *S. aureus* after cocultivation. **d** Photographs of agar plates with bacterial colony forming units of *S. aureus* and *E. coli*) in terms of blank control, PTMAO, PAP, and PTAP2, respectively. **e** SEM images of the surfaces of PTMAO, PAP, and PTAP2 after cocultivation. **f** Optical microscopy images of Chlorella adhesion on the surfaces of blank control, PTMAO, PAP, and PTAP2 after coincubation

In real seawater environments, the primary interfering substances include not only various salt ions, but also a wide spectrum of microorganisms, such as proteins, bacteria, and algae. Microbial adhesion and proliferation at the photothermal interface compromise the structural integrity of materials, thereby reducing their cycling stability. Beyond its excellent salt resistance, the nonfouling performance of PTAP2 was evaluated against diverse contaminants, including BSA, two representative bacteria (*E. coli* and *S. aureus*), and three types of algae (Chlorella, Diatoms and Chrysophytes) [55]. In the anti-protein adhesion test, the amount of BSA adsorbed on the PTAP2 surface is only 0.61 mg cm^−2^, comparable to that on PTMAO (0.55 mg cm^−2^) but markedly lower than that on PAP (1.81 mg cm^−2^) (Figs. 5a and S36). This superior performance is attributed to the overall electrostatic neutrality of zwitterionic PTMAO, which minimizes interactions with negatively charged BSA. In addition, the strong hydration capability of PTMAO facilitates the formation of a dense and continuous superhydrated shell on the PTAP2 surface, serving as an effective physical barrier that prevents BSA adsorption.

In the bacterial adhesion assay, the OD_600 nm_ values of bacterial suspensions in the PAP co-culture group decrease significantly (0.478 for *E. coli* and 0.481 for *S. aureus*) compared to the control group (0.640 for *E. coli* and 0.678 for *S. aureus*), indicating substantial bacterial adhesion on the PAP surface. In contrast, the OD_600 nm_ values for PTAP2 (0.589 for *E. coli* and 0.621 for *S. aureus*) approach those of the control, suggesting reduced bacterial adhesion attributed to the incorporation of PTMAO (Fig. 5b, c) [35]. Anti-bacterial adhesion performance is further evaluated by co-culturing hydrogels with *E. coli* and *S. aureus*. Dense colony formation is observed on agar plates from the original bacterial suspensions. Plates co-cultured with PAP display a reduced, yet still notable, number of colonies compared to the blank control. PTMAO- and PTAP2-cultured plates exhibit almost no detectable colonies (Fig. 5d). Consistently, SEM imaging reveals extensive bacterial adhesion on PAP surfaces, while PTAP2 surfaces remain virtually free of bacteria, confirming its superior anti-adhesion properties and its ability to mitigate biofilm-induced material degradation (Fig. 5e). Similarly, PTAP2 exhibits excellent anti-algal adhesion properties. Compared to the blank control and PAP surface, the density of Chlorella, Diatoms and Chrysophytes on the PTAP2 surface is markedly lower, approaching the minimal levels observed for PTMAO (Figs. 5f and S37). Collectively, these results demonstrate that the designed PTAP2, through the incorporation of PTMAO, forms a stable and continuous superhydrated shell, conferring robust nonfouling performance and enhanced durability in complex marine environments rich in proteins, bacteria, and algae, thus rendering it highly promising for efficient SID applications.

### Outdoor Water Collection Using Actual Seawater

**Fig. 6:**
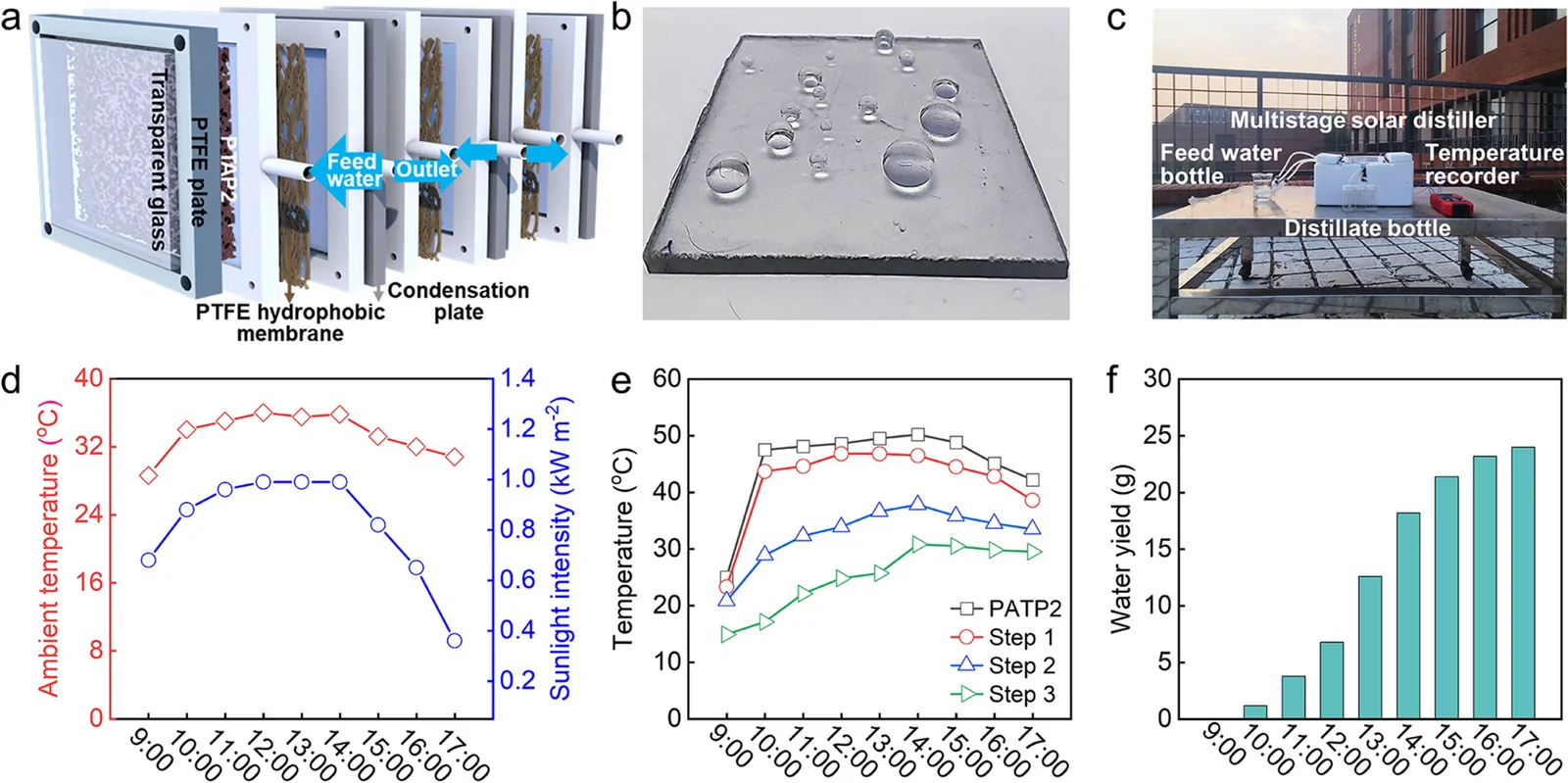
**a** Schematic diagram of the multistage solar distiller, including transparent glass cover, polytetrafluoroethylene (PTFE) support plate, PTAP2, feed water, PTFE hydrophobic membrane, hydrophobically modified condensation plate and outlet, respectively. **b** Photograph of transparent glass surface after hydrophobic modification. **c** Photograph of the multistage solar distiller during long-time outdoor testing. **d** Recorded curves of ambient temperature and sunlight intensity under natural conditions. **e** Temperature variation curves of the PTAP2 surface and each condensation stage. **f** Water yield of the multistage solar distiller versus time

Benefiting from the strong hydration capability, PTAP2 exhibits excellent water evaporation performance, as well as remarkable salt resistance and nonfouling properties. PTAP2 was incorporated into a designed multistage solar distiller, and long-term water collection experiments were conducted using actual seawater from the Bohai Sea (Fig. 6a–c and S38). To enhance the condensation of water vapor, the surfaces of the transparent glass and the condensation plates were treated to be superhydrophobic, thereby promoting efficient water vapor condensation and liquid water harvesting (Fig. S39). During outdoor testing, key parameters including ambient temperature, sunlight intensity, surface temperature (PTAP2 and each condensation stage), and the collected water amount were systematically recorded (Fig. 6d–f). The PTAP2-based multistage solar distiller achieved a water yield of 25.6 kg m^−2^ day^−1^ under natural environmental conditions. Moreover, no salt accumulation or microbial growth is observed on the PTAP2 surface after 60 continuous days of operation, demonstrating its long-term applicability in real seawater environments.

## Conclusion

In summary, we have developed a superhydrated zwitterionic PTAP with dedicated water channels for efficient, durable, and nonfouling SID under harsh marine conditions. Benefiting from the superhydrated ability of PTMAO, PTAP exhibits robust salt resistance and superior fouling inhibition against various foulants including proteins, bacteria, and algae. Molecular dynamics simulations reveal that the extraordinary superhydrated shell fundamentally governs its nonfouling behavior. This work highlights a biomimetic strategy to engineer superhydrated interfaces and paves the way for next-generation HSEs capable of stable and sustainable desalination in real-world marine environments. Looking forward, the molecular design principles demonstrated here could inspire the development of advanced HSEs for a broader range of water purification and marine applications.

## Supplementary Information

Below is the link to the electronic supplementary material.

Supplementary file1 (DOCX 17196 KB)
