# Supplementary material for: Superhydrated Zwitterionic Hydrogel with Dedicated Water Channels Enables Nonfouling Solar Desalination
Source: Nanomicro Lett. 2025 Dec 8;18:87. doi: 10.1007/s40820-025-01937-4 (PMC12682730; doi:10.1007/s40820-025-01937-4)
Supplement: Supplementary file 1 — Supplementary file1 (DOCX 17196 KB) [file 40820_2025_1937_MOESM1_ESM.docx]

Supporting Information for

**Superhydrated Zwitterionic Hydrogel with** **Dedicated Water Channels Enables Nonfouling Solar Desalination**

Panpan Zhang^1,*#^, Hanxue Liang^2#^, Yawei Du^1#^, Haiyang Wang^1^, Yaqi Tian^1^, Jingtao Bi^1^, Lei Wang^1^, Zhiyuan Guo^1^, Jing Wang^1^, Zhi-Yong Ji^1,*^, Liangti Qu^3,*^

^1^Engineering Research Center of Seawater Utilization Technology of Ministry of Education, Hebei Collaborative Innovation Center of Modern Marine Chemical Technology, Tianjin Key Laboratory of Chemical Process Safety, School of Chemical Engineering and Technology, Hebei University of Technology, Tianjin 300130, P. R. China

^2^Research Center for Analytical Sciences, Tianjin Key Laboratory of Biosensing and Molecular Recognition, College of Chemistry, Nankai University, Tianjin 300071, P. R. China.

^3^Key Laboratory of Organic Optoelectronics & Molecular Engineering, Ministry of Education, Department of Chemistry, Tsinghua University, Beijing 100084, P. R. China

^#^Panpan Zhang, Hanxue Liang, and Yawei Du contributed equally to this work.

***Corresponding authors. E-mail: [zhangpanpan@hebut.edu.cn](mailto:zhangpanpan@hebut.edu.cn) (Panpan Zhang); [jizhiyong@hebut.edu.cn](mailto:jizhiyong@hebut.edu.cn) (Zhi-Yong Ji); [lqu@mail.tsinghua.edu.cn](mailto:lqu@mail.tsinghua.edu.cn) (Liangti Qu)

**S1 Experimental Section**

*Chemicals and Materials*: Diethylenetriaminepentaacetic acid (AR, 99%)，2-Hydroxy-2-methylpropiophenone (Photoinitiator 2959, ≥97%) and phosphate buffer saline were supplied by Shanghai Aladdin Biochemical Technology Co., Ltd. N,N-Dimethylaminopropyl acrylamide (DMAPAA, 98%), Acrylamide (AAm, AR, 99%), N,N’-methylenebisacrylamide (MBAA, 99.7%), ammonium persulfate ((NH_4_)_2_S_2_O_8_, 99.99%), Pyrrole (Py, 99%), Phytic acid (IP6, 50wt%), were purchased from Shanghai Macklin Biochemical Technology Co., Ltd. Hydrogen peroxide (H_2_O_2_, 30wt%) was supplied by Tianjin Fuchen Chemical Reagent Co. Ltd. Dichloromethane (CH_2_Cl_2_, AR, ≥99.5%) and Sodium Chloride (NaCl, AR, ≥99.5%) were purchased from Tianjin Kermel Chemical Reagent Co. Ltd. Luria-Bertani (LB) Broth and Plate count agar were purchased from Qingdao Hope Bio-Technology Co., Ltd. Chlorella powder, Diatoms and Chrysophytes solutions were supplied by Qingdao Kangyuan Microalgae Biochemical Co., Ltd. Bovine serum albumin (BR, 98%) was supplied by Shanghai Yuanye Bio-Technology Co., Ltd. Deionized (DI) water was used throughout the experiment unless particularly stated.

*Synthesis of TMAO* *Monomer*: The zwitterionic monomer TMAO was synthesized via a one-step oxidation process [S1]. Briefly, 200 mg of diethylenetriaminepentaacetic acid (DTPA) was fully dissolved in 15 mL of deionized water, followed by the slow addition of 10 mL of 30% hydrogen peroxide (H_2_O_2_) solution. The mixture was subsequently heated to 60 °C and transferred into a three-necked flask pre-filled with oxygen (O_2_). Separately, 3.6 g of N,N-dimethylaminopropyl acrylamide was completely dissolved in 10 mL of deionized water and added dropwise into the flask. The reaction was maintained at 60 °C under continuous stirring for 6 h. Upon completion, the mixture was cooled to room temperature, and the resulting product was extracted multiple times with dichloromethane. The final product was obtained as a colorless, viscous TMAO monomer by freeze-drying.

*Preparation of PTAPs*: Various amounts of TMAO (100, 200, 300, and 400 mg), AAm (560 mg), the cross-linker MBAA, and the photoinitiator 2959 were dissolved in 4 mL of deionized water. The concentrations of MBAA and 2959 were set at 1.5 wt% and 0.15 wt% relative to the mass of TMAO, respectively. The mixture was homogenized by ultrasonication and subsequently photopolymerized under UV irradiation for 30 min to form PTMAO/PAAm hydrogels. The as-prepared hydrogels were immersed in deionized water overnight to achieve full swelling, followed by transfer into 10 mL of deionized water containing 69 μL of pyrrole and 1 mL of phytic acid for 24 h. In situ polymerization was then conducted in a 0.1 mol L^−1^ ammonium persulfate solution for 1 h. Residual pyrrole monomers were removed by extensive washing with deionized water, yielding PTMAO/PAAm/PPy hydrogels (denoted as PTAPs). The hydrogels were labeled PTAP1, PTAP2, PTAP3, and PTAP4 corresponding to TMAO contents of 100, 200, 300, and 400 mg, respectively.

*Fabrication of PTP and PAP Hydrogels*: Typically, 400 mg of TMAO monomer, 60 mg of the cross-linker N,N’-methylenebisacrylamide (MBAA), and 6 mg of the photoinitiator 2-hydroxy-2-methylpropiophenone (2959) were added to 2 mL of deionized water. The mixture was homogenized by ultrasonic dispersion in a sonicator bath, followed by UV-induced photopolymerization for 30 min to form the PTMAO hydrogel. In a separate preparation, 420 mg of acrylamide (AAm), 63 mg of MBAA, and 6.3 mg of 2959 were dissolved in 4 mL of deionized water and sonicated for homogeneous dispersion. The solution was then photopolymerized under UV light for 30 min to form the PAAm hydrogel. The resulting PTMAO and PAAm hydrogels were immersed in 10 mL of deionized water containing 69 μL of pyrrole and 1 mL of phytic acid for 24 h to ensure complete absorption of the pyrrole monomers. Subsequently, the hydrogels were placed in a 0.1 mol L^−1^ ammonium persulfate solution for in situ polymerization for 1 h. After polymerization, the gels were rinsed with deionized water and further soaked for 24 h. Finally, the residual pyrrole monomers were removed, yielding PTMAO/PPy (PTP) and PAAm/PPy (PAP) hydrogels.

*Mechanical Analysis*: The mechanical properties of fully hydrated cylindrical hydrogels (16 mm in diameter and 25 mm in height) were evaluated using a universal testing machine equipped with a 100 N load cell. Before compression testing, the specimens were dried on the surface, and their initial dimensions were measured. During the test, each sample was positioned vertically on the base platform, and the upper compression plate was aligned to establish zero displacement contact before initiating compression at a constant rate of 5 mm min^−1^. All measurements were conducted under controlled ambient conditions, with each specimen tested in triplicate. The final mechanical properties were determined by averaging three independent measurements, and the variability in the data was assessed through standard deviation.

*Melting and Evaporation Behavior Tests*: Differential scanning calorimetry (DSC) was employed to analyze the evaporation and ice-melting behaviors of the hydrogels. After removing surface water from the fully swollen hydrogels, samples (10–12 mg) were weighed and placed in sealed aluminum crucibles. For the evaporation test, the samples were heated from 25 °C to 140 °C at a rate of 5 °C min^–1^ in a nitrogen atmosphere, with evaporation enthalpy calculated by integrating the DSC curve. In the ice-melting analysis, the samples were first cooled to –30 °C at 5 °C min^–1^, maintained at this temperature for 10 min, and then heated to 30 °C at the same rate under nitrogen. The enthalpy of the endothermic peak was used to quantify the melting behavior.

*Solar-Driven Interfacial Desalination (SID) Experiments*: The SID device was designed with a three-layer configuration including a solar evaporator (20 mm × 20 mm active area), a polystyrene foam (PS) thermal insulation layer (10 mm thickness), and hydrophilic cotton rods embedded within the PS matrix to serve as water transport channels. The assembled device was submerged in a water reservoir, with the cotton rods ensuring a continuous water supply to the photothermal layer. The SID performance was evaluated under sunlight irradiation (1 kW m^−2^). Mass loss was monitored hourly using an analytical balance with a resolution of 0.1 mg to calculate the evaporation rate.

The rate of water evaporation (*v*) is calculated from the following equation [S2]:

| $\text{v}\text{=d}\text{m}\text{/(}\text{S}\text{∙d}\text{t}\text{)}$ | （S1） |
| --- | --- |

where *v* is the rate of water evaporation, *m* is the change in mass of the device during evaporation, *S* is the area of the solar evaporator under light irradiation, and *t* is the time.

The photothermal conversion efficiency (*η*) is calculated from the following equation [S2]:

| $\text{η=v}\text{h}_{\text{Lv}}\text{/(}\text{C}_{\text{opt}}\text{P}_{\text{0}}\text{)}$ | （S2） |
| --- | --- |

Where *h*_Lv_ is the evaporation enthalpy of water in the hydrogel, *C*_opt_ is the optical concentration, and *P*_0_ is the solar irradiation power of 1 kW m^–2^. For instance, PTAP2 has the lowest energy consumption with an evaporation enthalpy of 1450 J g^−1^, and delivers the highest water evaporation rate, reaching 2.28 kg m^−2^ h^−1^. As such, the energy utilization efficiency of PTAP2 is calculated as 91.8%, outperforming pure water, PAP, and other PTAPs.

A 10 mm × 10 mm hydrogel sample was cut from the fully swollen hydrogel. Surface moisture was blotted with filter paper, and the initial mass (*M*_1_) was recorded using a 0.1 mg-resolution analytical balance. The sample was then dried in an 80 °C oven until constant mass was achieved, cooled to room temperature in a desiccator, and reweighed to obtain the dry mass (*M*_0_). The equilibrium water content (*Q*_s_) was calculated according to the following Equation:

| $\text{Q}_{\text{s}}\text{=}\frac{\text{M}_{\text{1}}\text{-}\text{M}_{\text{0}}}{\text{M}_{\text{0}}}\text{×100\%}$ | （S3） |
| --- | --- |

*Anti-Fouling Performance Test*:

*Anti-Protein Measurements*: A standard calibration curve for the quantification of bovine serum albumin (BSA) adsorption was constructed by measuring the UV-vis absorbance at 280 nm for BSA solutions at varying concentrations [S3]. Hydrogel samples were first equilibrated in deionized water for 24 h, followed by immersion in a 0.5 mg mL^–1^ BSA solution for another 24 h. Subsequently, the samples were carefully extracted with tweezers and gently rinsed with phosphate-buffered saline (PBS) to eliminate weakly adsorbed BSA. They were then washed thoroughly with ultrapure water to remove strongly bound BSA. The absorbance of the eluates was recorded at λ = 280 nm using UV-vis spectroscopy and compared to the pre-established calibration curve to determine the surface-adsorbed BSA content.

*Anti-Bacterial Experiments*: Bacterial adhesion experiments were conducted with S. aureus and E. coli using primary cultures grown in lysogeny broth (LB) and incubated at 37 °C until reaching the exponential growth phase. Under a laminar flow hood, 0.1 mL of the bacterial culture was diluted with a 0.9% sterile NaCl solution to the desired concentrations, with the same saline solution used as a blank control. Absorbance at λ = 600 nm was measured to adjust the bacterial suspensions to an optical density (OD_600 nm_ = 0.6–0.7). Sterilized hydrogel samples were immersed in these bacterial suspensions and incubated at 4 °C for 24 h. After incubation, the optical density of the remaining bacterial suspension was assessed to determine the hydrogel’s anti-adhesion effectiveness. To prepare for antimicrobial experiments, the bacterial suspension was serially diluted with sterile medium to an optimal concentration (OD_600 nm_ = 0.20). Following standardized protocols, the diluted suspension was aseptically plated onto nutrient agar using a spiral plater and incubated at 37 °C for 24 h under aerobic conditions. The number of bacterial colonies on the agar plates was then counted to evaluate the antimicrobial properties of the hydrogel samples.

*Algae Resistance Evaluation*: A hydrogel sample (10 mm × 10 mm) was cut from the fully swollen and saturated hydrogel. The hydrogel was then immersed in a 2 mg mL^–1^ Chlorella, Diatoms and Chrysophytes solutions and co-cultured for 24 h. After incubation, the sample was carefully retrieved using tweezers and rinsed three times with deionized water. The distribution density of algae on the hydrogel surface was observed under an inverted biological microscope.

*Characterization*: The molecular structure of TMAO was analyzed using proton nuclear magnetic resonance spectroscopy (1H NMR, AVANCE 400, Bruker). The micromorphology of the hydrogels was observed with field emission scanning electron microscopy (FESEM, JSM-7610F, JEOL, Japan). Elemental composition and chemical bonding at the hydrogel surface were investigated by X-ray photoelectron spectroscopy (XPS, Escalab 250Xi, Thermo Scientific). Fourier-transform infrared (FTIR) spectroscopy (V80, Bruker) was employed to identify functional groups and chemical bonds within the hydrogels, with spectral scans conducted over the range of 4000–500 cm^–1^. Raman spectroscopy (inVia Reflex) provided further insight into the chemical bonding structure. Light absorption properties were examined using a UV-visible near-infrared spectrophotometer (Shimadzu UV-3600). Evaporation and ice-melting behaviors were assessed using differential scanning calorimetry (DSC, Hengjiu Corporation, HSC-4, China). Mechanical testing of the hydrogels was performed using a microcomputer-controlled universal testing machine (CMT6104). Surface temperature was recorded with both an infrared camera (Fotric 326) and a thermocouple temperature detector (TA612C).

**Supplementary Figures**

**
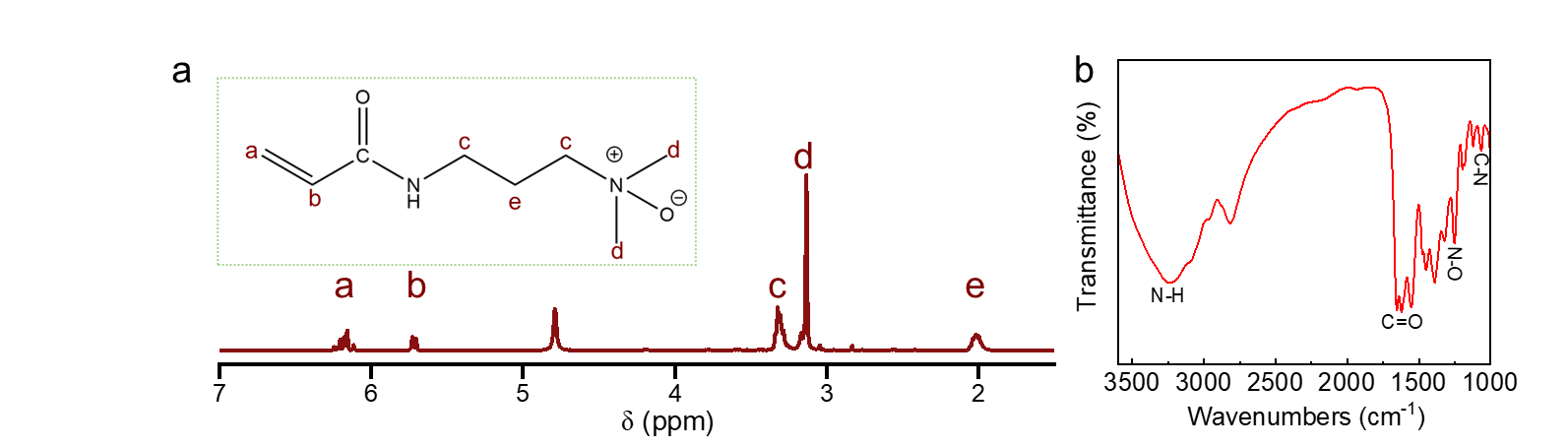
**

**Fig. S1** (**a**) 1H NMR spectra of the TMAO in D_2_O. (**b**) FTIR spectra of TMAO monomer

The material design draws inspiration from the adaptive strategies of saltwater fish, which maintain osmotic homeostasis and resist biofouling in hypersaline, microbe-rich environments. Notably, trimethylamine N-oxide (TMAO), a naturally occurring osmolyte in marine fish, serves as an ideal zwitterionic motif for nonfouling materials due to its directly linked oppositely charged groups (Me_3_N^+^–O^−^), which form strong hydration shells without the need for a spacer [S1].

The 1H NMR spectrum of the TMAO monomer reveals distinct peaks at 3.30 ppm and 3.12 ppm, which are attributed to the tertiary amine and methyl groups, respectively, confirming the oxidation of DMAPA to TMAO. FTIR analysis of TMAO shows a broad band at 3300 cm^−1^, corresponding to the N−H stretching vibration, and a peak at 1650 cm^−1^, which is linked to the C=O stretching vibration. Additional characteristic peaks at 1250 cm^−1^ and 1050 cm^−1^ are associated with the N–O and C–N stretching vibrations, respectively. These results validate the successful synthesis of the TMAO monomer.


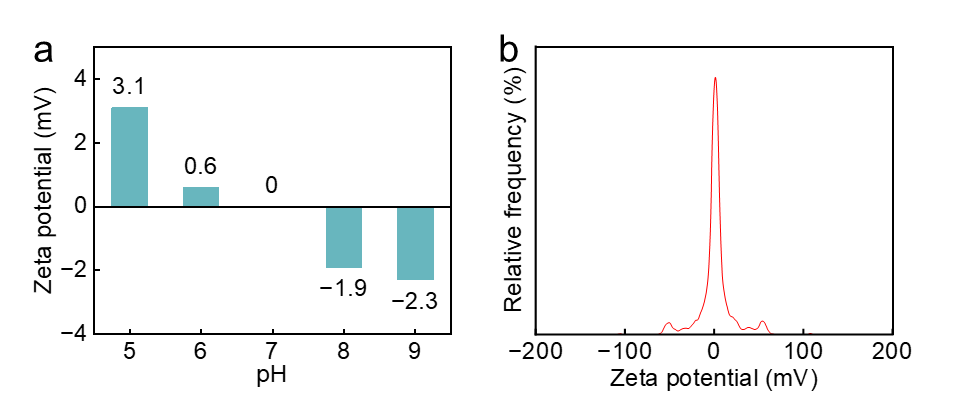


**Fig. S2** Zeta potential analysis of PTMAO (**a**) at different pH and (**b**) at pH=7.0

We have conducted zeta potential measurements of the PTMAO hydrogel surface. The measured zeta potential is 0 mV (pH=7), confirming that the hydrogel surface is nearly electrostatically neutral.

**
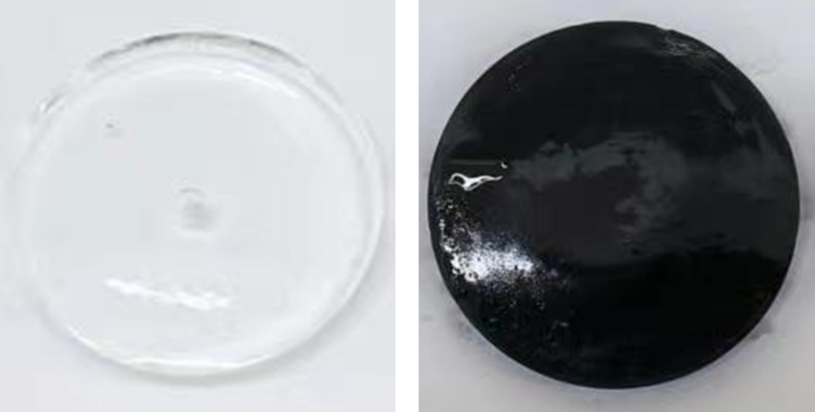
**

**Fig. S3** Photographs of PTAP2 hydrogel before and after loading of PPy

As one example, we use black PPy as the light-absorbing material. After immersing the white hydrogel in pyrrole monomer for crosslinking, a solar evaporator (PTAP2) with distinct black coloration is obtained, demonstrating excellent light absorption performance across the full spectrum of sunlight.


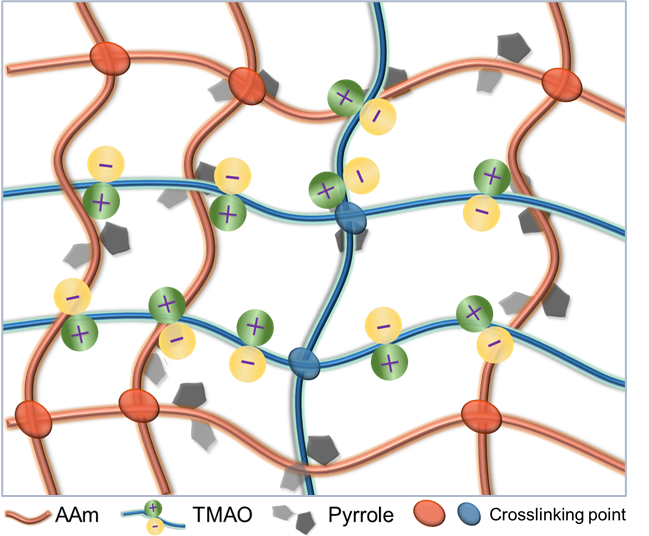


**Fig. S4** Schematic illustration of the formation mechanism of PTAP

The zwitterionic PTMAO carries directly linked positively charged N^+^ and negatively charged O^−^ groups. The O^−^ can form strong bonding interactions with water molecules, allowing them transport to the photothermal interface for fast evaporation. Simultaneously, the N^+^ can strongly repels salt ions and prevents damage to the superhydrated shell, ensuring exceptional nonfouling performance in continuous SID process. Nevertheless, the pure PTMAO hydrogel exhibits poor mechanical strength, which limits its long-term applicability. To overcome this limitation, PAAm was incorporated into the polymeric network to reinforce the mechanical robustness and enhance the durability of PTAP. The superior mechanical performance of PAAm arises from its flexible carbon backbone, the abundance of amide groups capable of forming hydrogen bonds, and its excellent compatibility for crosslinking and hybridization. These characteristics make PAAm widely utilized as a structural enhancer and toughening agent in hydrogel and soft material systems [S4]. Additionally, polypyrrole (PPy), a black conductive polymer, serves as an efficient photothermal agent that converts solar radiation into heat for water evaporation. Within the PTAP polymeric network, PAAm forms a mechanically resilient framework, PTMAO contributes to strong hydration through zwitterionic interactions, and PPy enhances light-to-heat conversion. The integrated design and formation mechanism of the PTAP system are illustrated as Fig. S4.


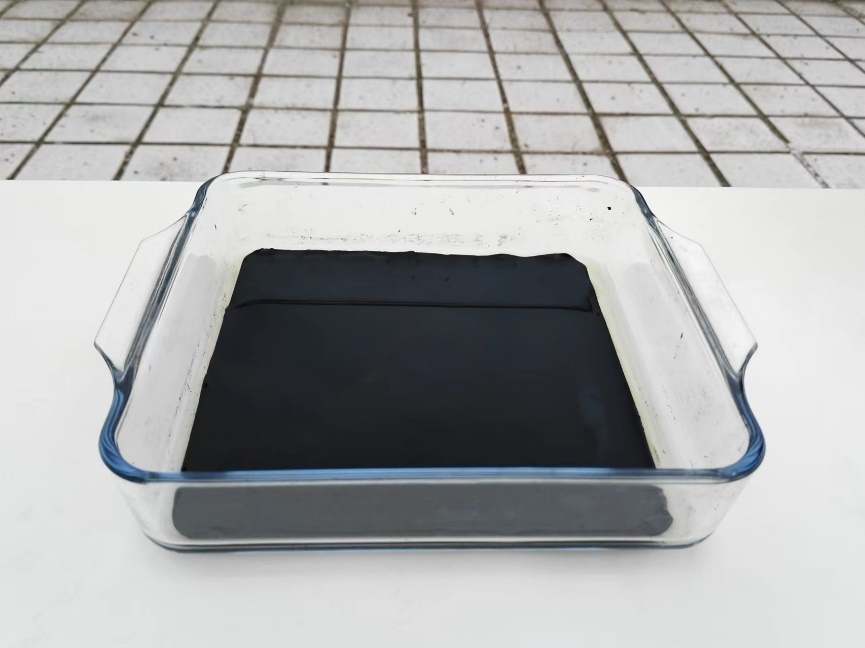


**Fig. S5** Photograph of the fabricated PTAP2 at large scale


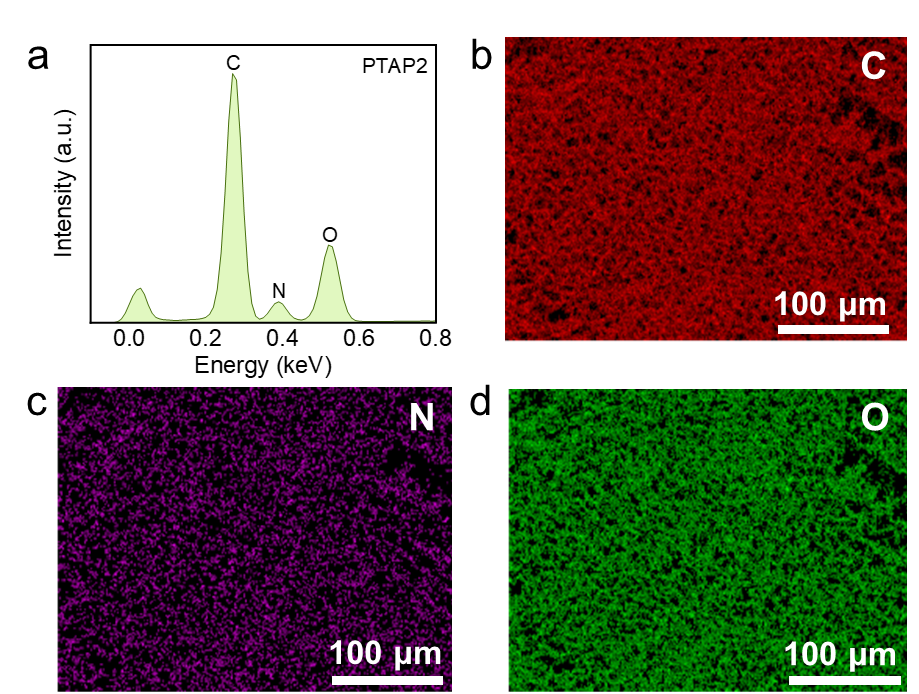


**Fig. S6** (**a**) EDS and (**b–d**) corresponding elemental mapping images of PTAP

We further conducted energy-dispersive X-ray spectroscopy (EDS) and elemental mapping to complement the characterization of PTAP. EDS results confirm the presence of C, N, and O as the primary elements within the PTAP. Corresponding elemental mapping images reveal their uniform distribution throughout the microstructure of PTAP.

**
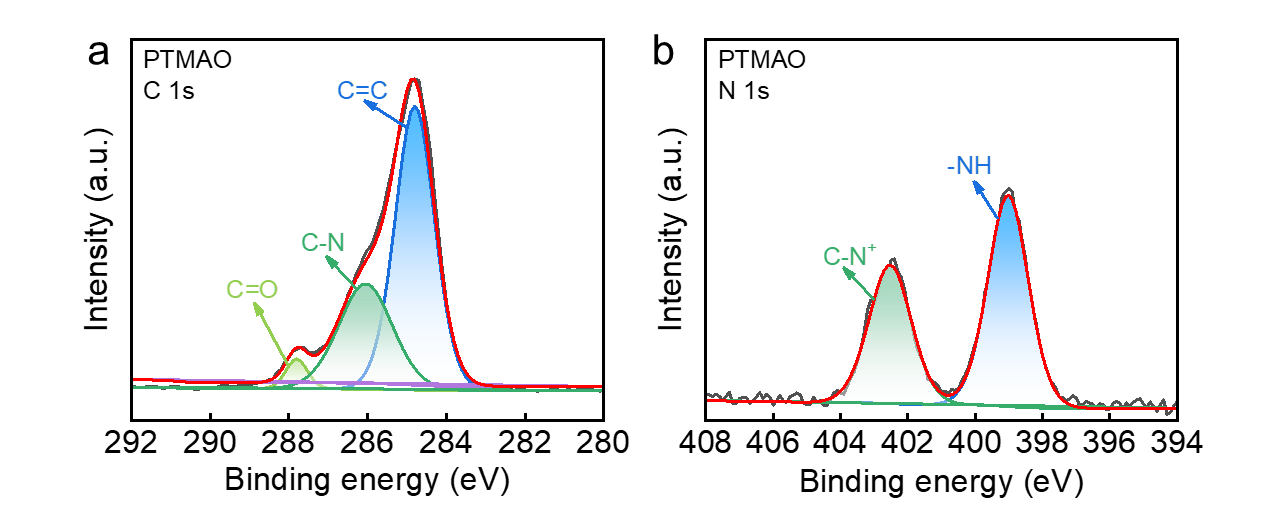
**

**Fig. S7** High-resolution (**a**) C 1s, and (**b**) N 1s XPS spectra of PTMAO

In the C 1*s* XPS spectrum of PTMAO hydrogel, three characteristic peaks are observed at 284.8, 286.0, and 287.9 eV, corresponding to the C–C, C−N, and C=O bonds, respectively. In the N1s XPS spectrum of PTMAO, peaks at 399.0 eV and 402.6 eV correspond to the –NH and C−N^+^ bonds, respectively.

**
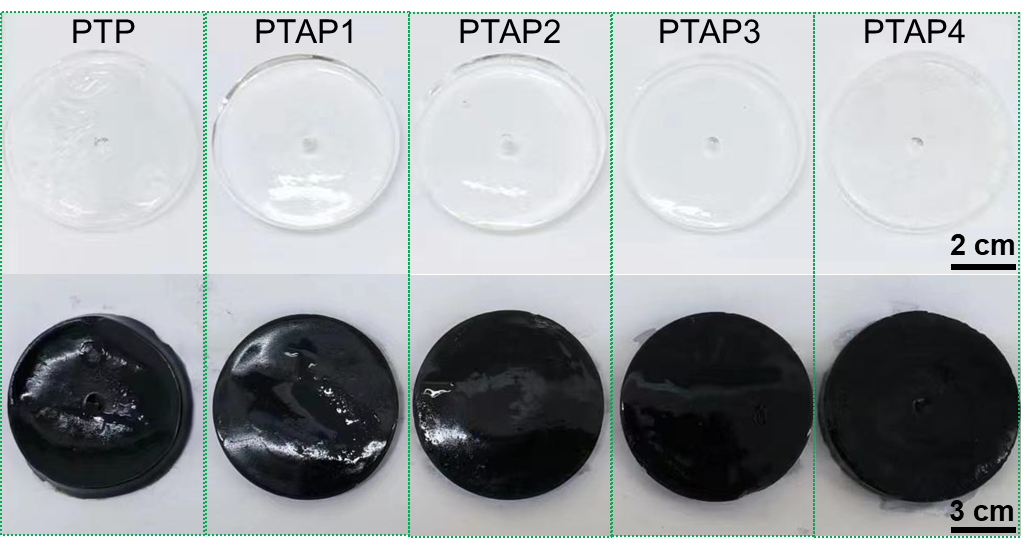
**

**Fig. S8** Photographs of PTP, PTAP1, PAP2, PTAP3 and PTAP4 hydrogels before and after PPy loading

It is worth noting that the mechanical properties of pure PTMAO hydrogels are poor, and further increase in the content of PTMAO will gradually weaken the mechanical properties of PTAP. Therefore, the content of PTMAO must be optimized to balance the mechanical properties of PTAP with the solar-driven water evaporation performance. To this end, we prepared PTAPs with different PTMAO contents: 100, 200, 300, and 400 mg of PTMAO per 560 mg of AAm, and named PTAP1, PTAP2, PTAP3, and PTAP4, respectively. The black PPy is incorporated into the hydrogel through an in-situ polymerization method. As shown in the photographs, PTP, PTAP1, PTAP2, PTAP3, and PTAP4 all exhibit a distinct black color, which enable excellent light absorption properties for SID experiments.

**
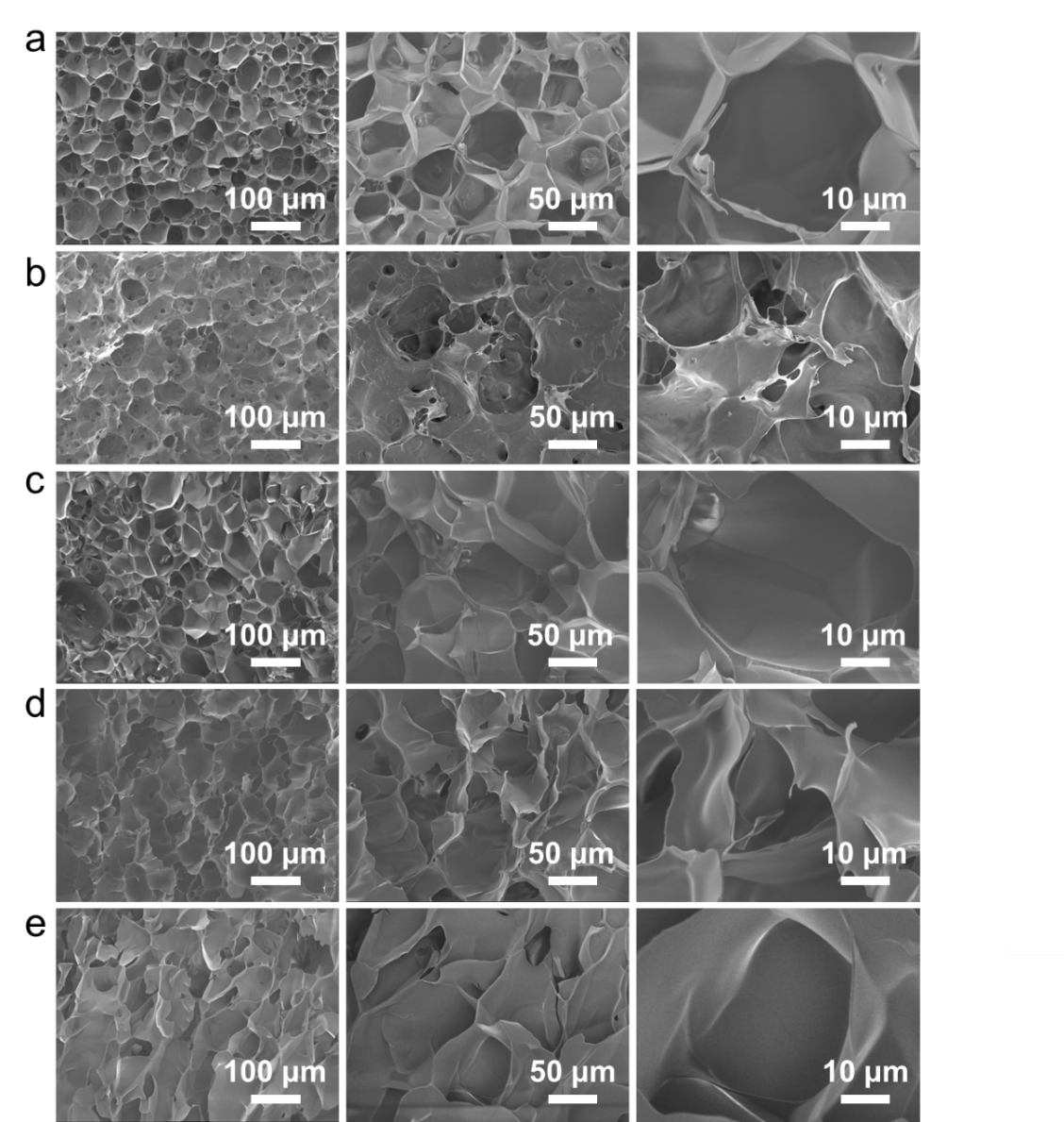
**

**Fig. S9** SEM images of (**a**) PAP, (**b**) PTAP1, (**c**) PTAP2, (**d**) PTAP3, and (**e**) PTAP4, respectively

SEM images indicate that the PAP hydrogel exhibits a uniform micrometer-scale porous network structure. After the introduction of the PTMAO polymer network, the PTAPs maintain their original micrometer-scale porosity, demonstrating that PTMAO and PAAm polymers can effectively copolymerize. The micrometer-scale porous structure in the PTAPs facilitates light absorption and rapid water transport, promoting excellent SID performance.


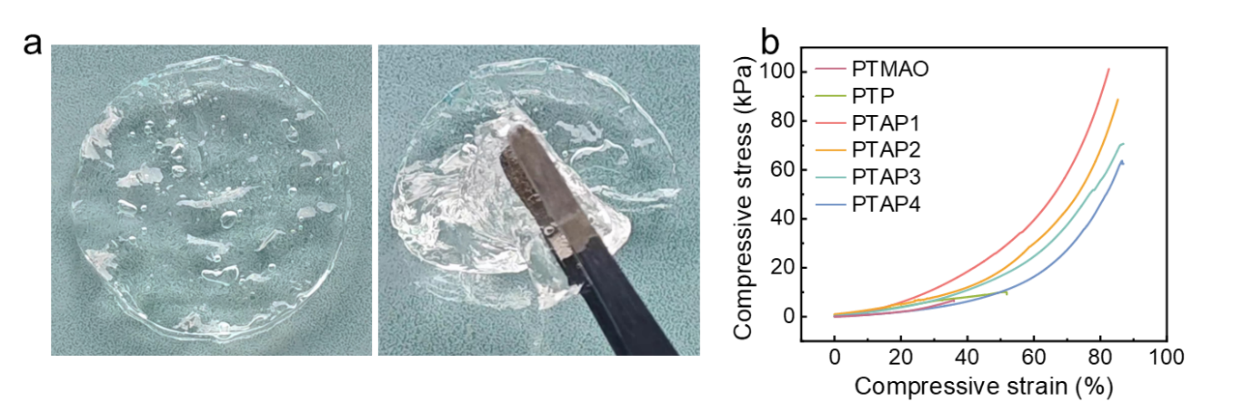


**Fig. S10** (**a**) Photographs of pure PTMAO hydrogel showing poor mechanical property. (**b**) Stress-strain curves of PTMAO, PTP and PTAP under compression after immersion in seawater

Zwitterionic PTMAO carries positively charged N^+^ and negatively charged O^−^ groups. The O^−^ can form strong bonding interactions with water molecules, allowing them transport to the photothermal interface for fast evaporation. Simultaneously, the N^+^ can strongly repels salt ions and prevents damage to the superhydrated shell, ensuring exceptional nonfouling performance in continuous SID process. However, the pure PTMAO hydrogel exhibits inherently poor mechanical strength, limiting its long-term applicability. To address this limitation, we incorporated PAAm to reinforce the network, thereby enhancing its durability and practical utility. As suggested, we have included the mechanical performance of the pure PTMAO hydrogel as a reference. The pure PTMAO hydrogel is mechanically fragile, showing obvious damage when lifted with tweezers. The compression test further confirms its weakness, with fracture occurring at a compressive strain of 35.8% and a stress of only 6.67 kPa. The copolymerization of PTMAO and PAAm polymer networks imparts excellent mechanical properties to the PTAP hydrogels. All PTAPs exhibit outstanding compressive strength in seawater environments, significantly surpassing the compressive resistance of the pure zwitterionic hydrogel PTP. However, as the PTMAO content increases, the mechanical properties of the PTAPs tend to deteriorate. Therefore, in subsequent work, we will optimize the selection of PTAPs based on both mechanical and SID performance.


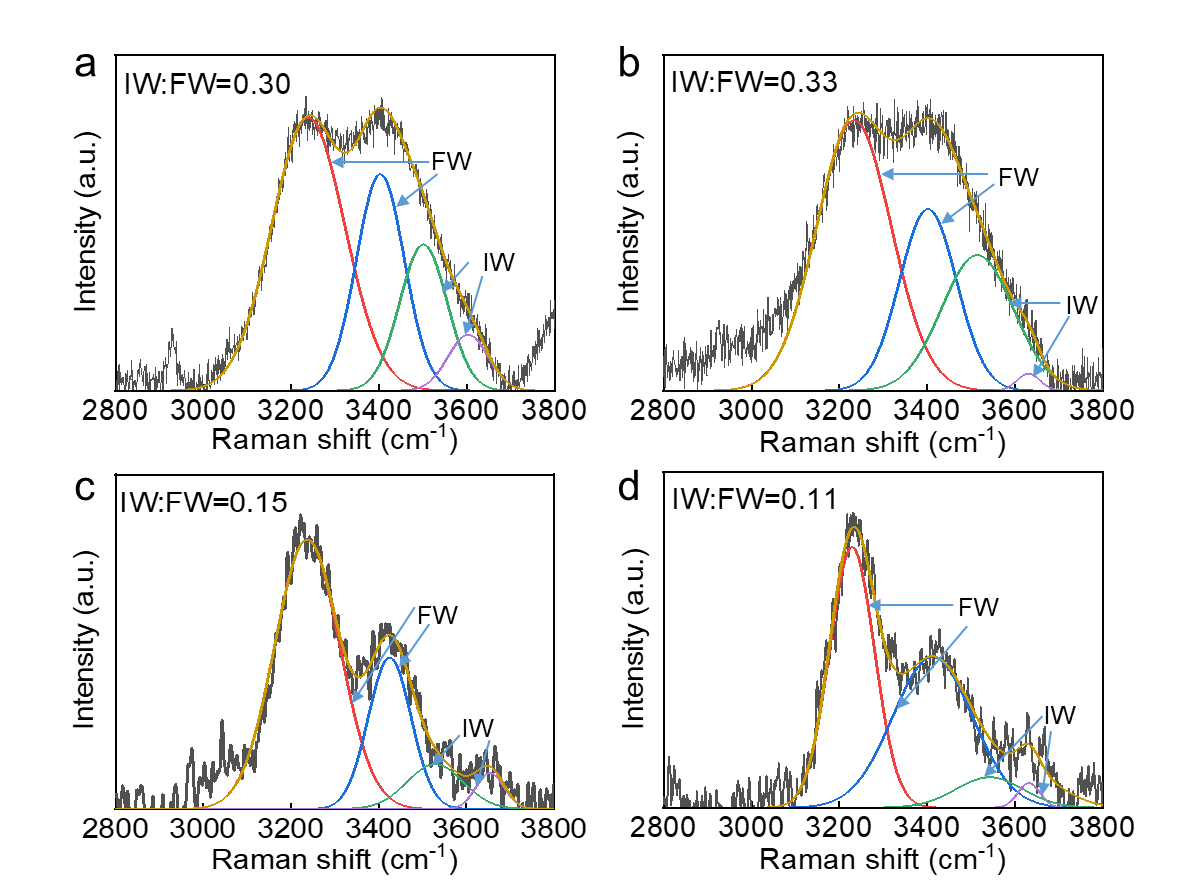


**Fig. S11** Raman spectra with fitting curves showing FW and IW in (**a**) PTAP1, (**b**) PTAP2, (**c**) PTAP3, and (**d**) PTAP4

Zwitterionic PTMAO that are entirely superhydrophilic are able to from very strong interactions with water, and thus regulating water states including bound water (BW), intermediate water (IW), and free water (FW) in PTAP. The Raman spectra reveal characteristic peaks at 3233, 3401, 3514, and 3630 cm^–1^, which are associated with the water states within the hydrogels. The peaks at 3233 and 3401 cm^–1^ arise from the heterogeneous O−H stretching modes, corresponding to FW. The peaks at 3514 and 3630 cm^–1^ are attributed to the symmetric and asymmetric stretching of weak hydrogen-bonded water molecules, representing IW. The IW:FW ratios in PTAP1, PTAP2, PTAP3, and PTAP4 are 0.3, 0.33, 0.15, and 0.11, respectively, with PTAP2 exhibiting the highest IW:FW ratio. The introduction of superhydrophilic PTMAO enables strong hydrogen bonding with water molecules, regulating the water states within PTAPs. From PAP to PTAP2, the IW content in these hydrogels gradually increase as the PTMAO content rise up. However, for PTAP3 and PTAP4, with further increases in PTMAO content, the degree of cross-linking is high and more water molecules are BW, resulting in a decrease in the IW:FW ratio.


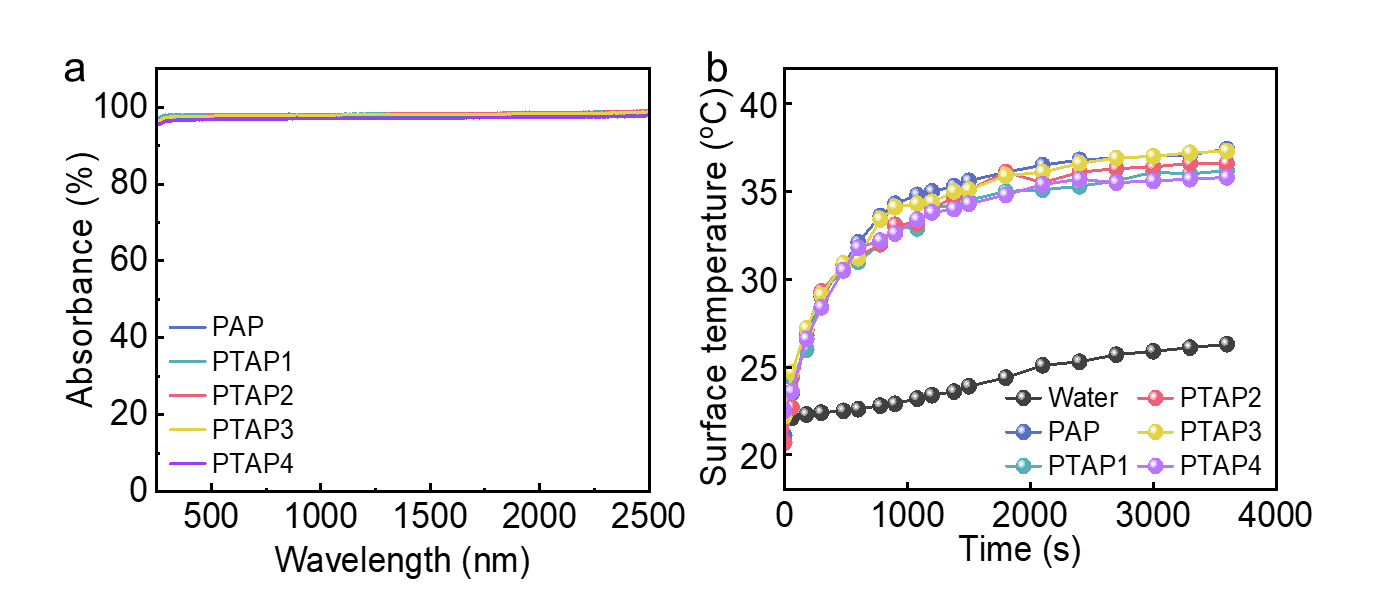


**Fig. S12** (**a**) Absorption spectra of PAP and PTAPs in the wavelength range of 250–2500 nm. (**b**) Surface temperature of PAP and PTAPs versus time

To enhance the light absorption properties of PAP and PTAPs, PPy is incorporated as a light-absorbing material through in-situ polymerization within the hydrogels. Across the wavelength range of 250–2500 nm, both PAP and PTAPs exhibit remarkable light absorption, with absorbance exceeding 98.5%. Under solar irradiation of 1 kW m^–2^, the surface temperatures of PAP and PTAPs rise rapidly within 10 min, stabilizing in the range of 36.0–37.5 °C, significantly higher than that of bulk water. The temperature difference between the hydrogel surfaces and the bulk water reaches up to 10 °C, demonstrating that the incorporation of PPy substantially enhances both the light absorption and photothermal conversion efficiency of PAP and PTAPs.


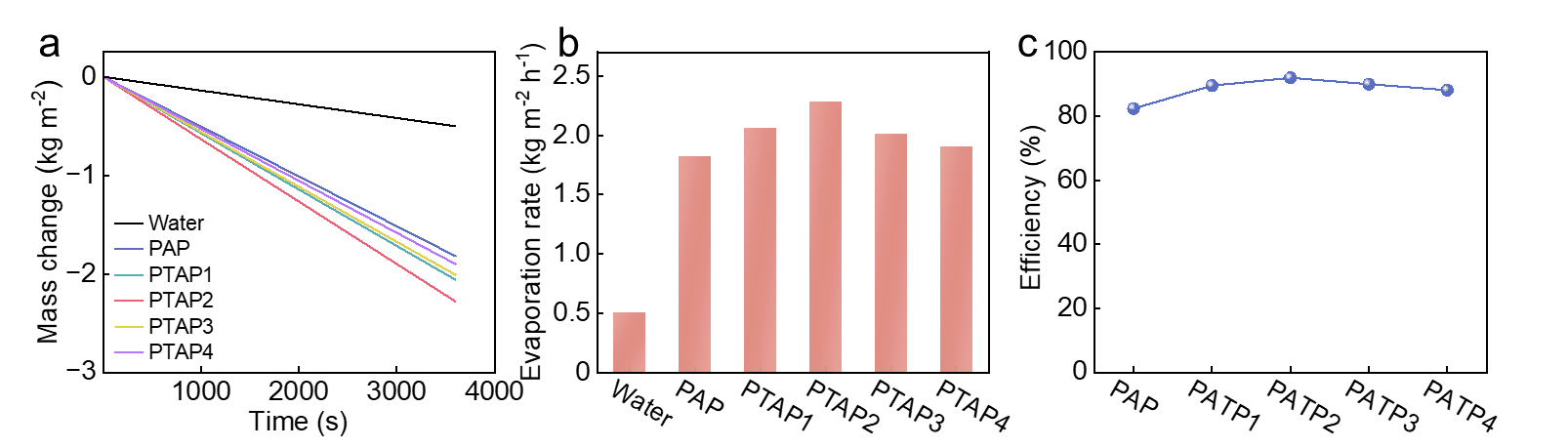


**Fig. S13** (**a**) Mass change, and (**b**) Evaporation rate of Water, PAP, and PTAPs; (**c**) Photothermal conversion efficiency of PAP and PTAPs

Under the solar irradiation of 1 kW m^–2^, the water evaporation rates of PTP, PTAP1, PAP2, PTAP3, and PTAP4 are 1.82, 2.06, 2.28, 2.01, and 1.9 kg m^−2^ h^−1^, respectively, significantly higher than the evaporation rate of pure water (0.50 kg m^−2^ h^−1^). Among them, PTAP2 exhibits the highest water evaporation rate, as the hydrophilic groups (O^−^) in the PTMAO polymer network activate water molecules, increasing the content of IW and reducing the energy demand during the solar driven water evaporation process. The water evaporation enthalpies of PTP, PTAP1, PAP2, PTAP3, and PTAP4 are 1630, 1565, 1450, 1612, and 1670 J g^−1^, respectively. The corresponding photothermal conversion efficiencies are 82.4%, 89.5%, 91.9%, 90.0%, and 88.1%, respectively. Notably, PTAP2 delivers the highest water evaporation rate, reaching 2.28 kg m^−2^ h^−1^, with an energy utilization efficiency of 91.8%, outperforming pure water, PAP, and other PTAPs.
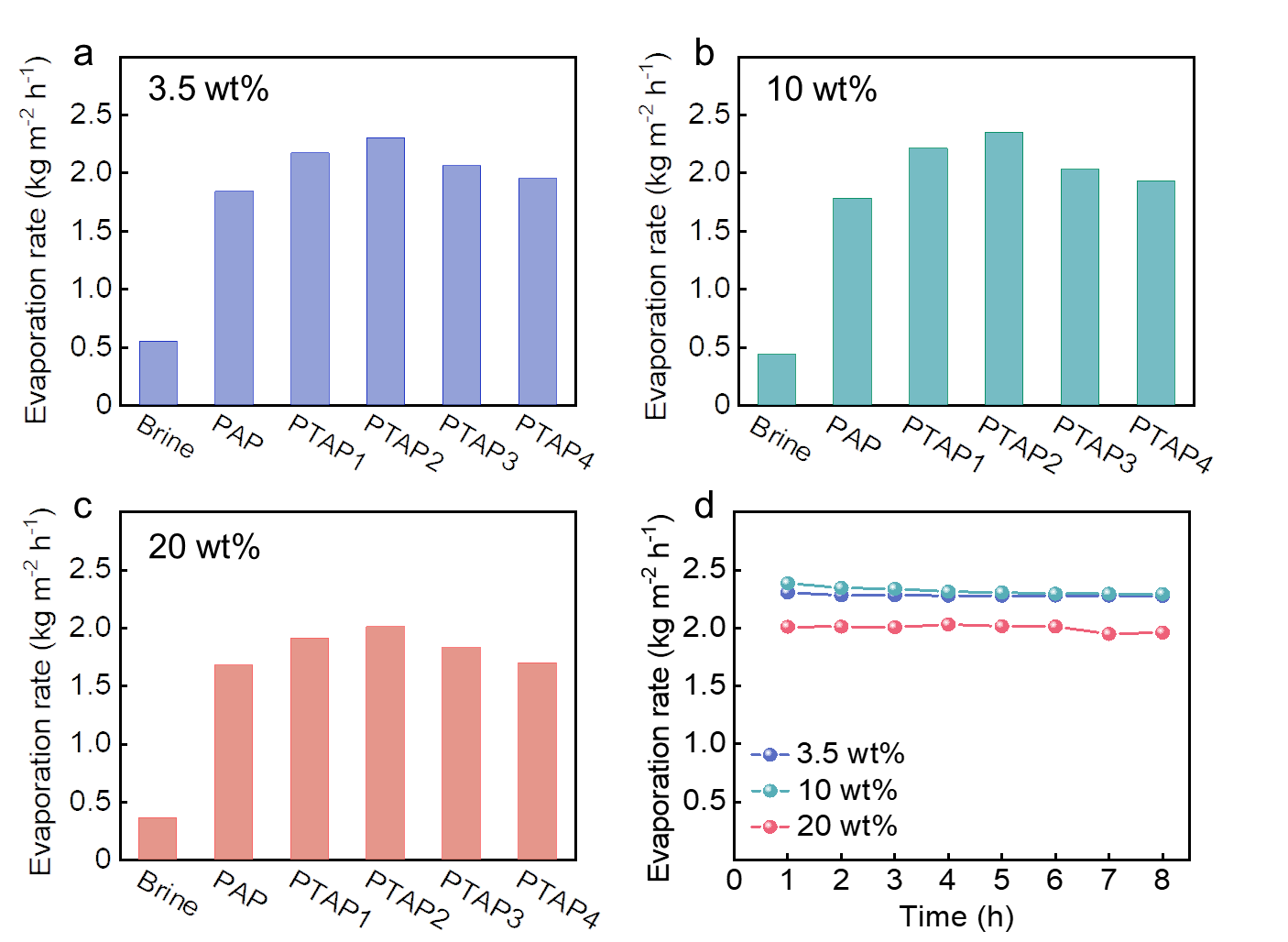


**Fig. S14** Evaporation rates of PAP and PTAPs in (**a**) 3.5 wt%, (**b**) 10 wt%, and (**c**) 20 wt% brines. (**d**) Long-time evaporation rate of PTAP2 in water with different salt concentrations

The long-term stability of water evaporation performance using PAP and PTAPs were systematically evaluated in brines with varying salinities (3.5 wt%, 10 wt%, and 20 wt%). The results demonstrate that PTAP2 exhibits the best water evaporation rates of 2.30, 2.35, and 2.01 kg m^−2^ h^−1^ in 3.5 wt%, 10 wt%, and 20 wt% brines under the solar irradiation of 1 kW m^−2^, respectively. Notably, as the salt concentration increases, the water evaporation rate of PTAP2 initially increases, peaking at 10 wt% brine, before declining. This trend is likely attributed to ionic screening effects at higher concentrations, which neutralize the charges on the zwitterionic polymer and reduce electrostatic interactions. As a result, the polymer network weakens, facilitating more efficient water transport to the evaporation interface. The long-term stability of PTAP2’s water evaporation performance is systematically evaluated in brines with varying salinities (3.5 wt%, 10 wt%, and 20 wt%) to assess its salt resistance. Under the solar irradiation of 1 kW m^−2^, continuous 8 h water evaporation tests are conducted in NaCl solutions with concentrations of 3.5 wt%, 10 wt%, and 20 wt%. The water evaporation rates of PTAP2 show no significant decrease, and no salt crystallization is observed on its surface, further demonstrating that PTAP2 possesses excellent stability and salt resistance.


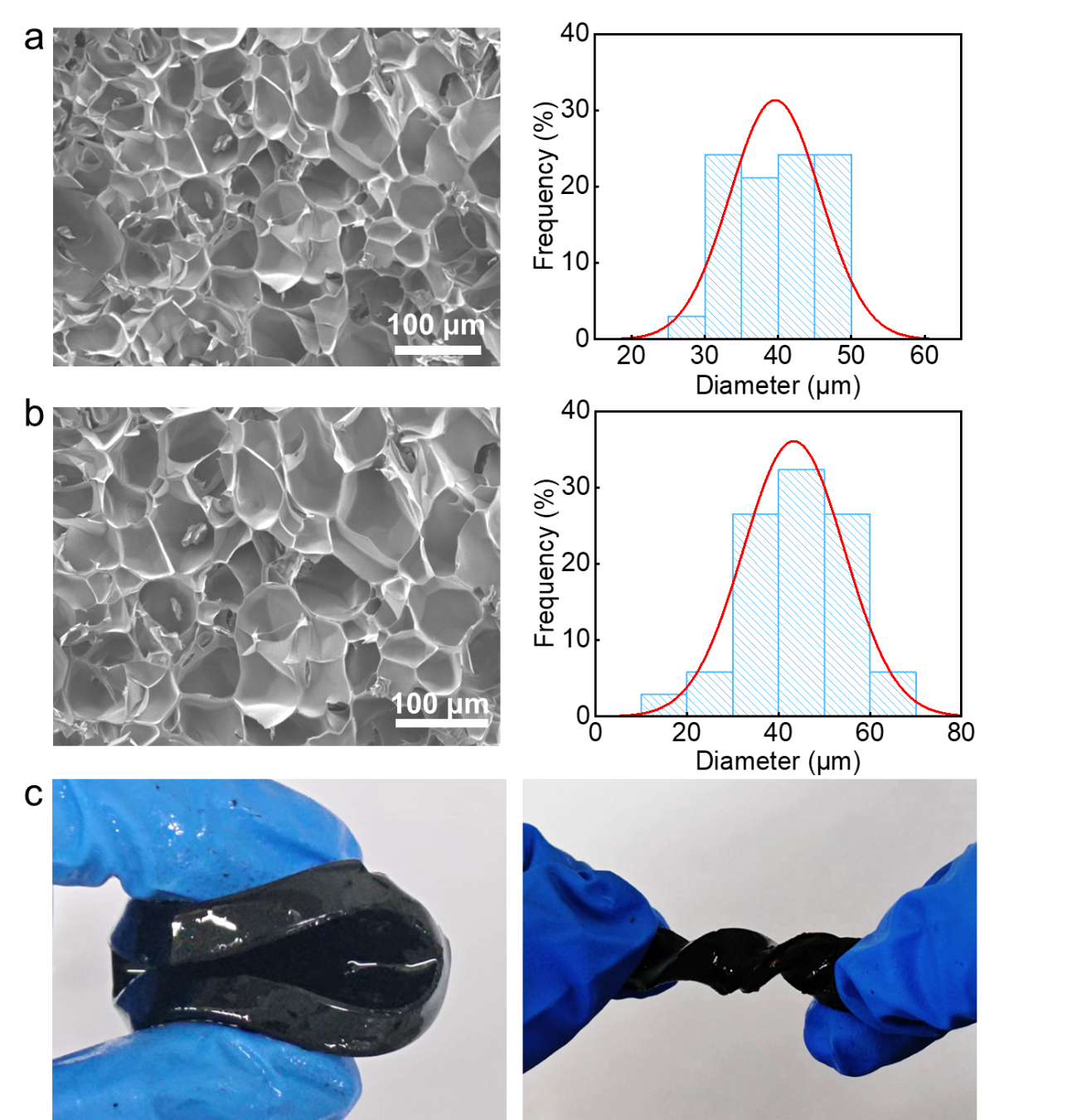


**Fig. S15** SEM image and corresponding pore size distribution curve of PTAP2 (**a**) before and (**b**) after exposure to 10 wt% NaCl solution. (**c**) Photograph of PTAP2 undergoing folding, and twisting after use in high-salinity solution (10 wt% NaCl)

At elevated salinities, ionic screening in the solution attenuates the intrinsic positive and negative charges of the zwitterionic polymer, thereby weakening electrostatic interactions between polymer chains. This reduction in intermolecular attraction relaxes the hydrogel network structure, enhancing water mobility toward the evaporation interface. This observation is consistent with previously reported findings [5]. To further investigate the effect of high salinity, we conducted SEM images and porosity analysis before and after exposure to 10 wt% NaCl solution. The results reveal that the pore sizes become bigger following use in high-salinity condition, indicating a relaxation of the PTAP2 network. Importantly, no visible cracks or structural damage are observed on the surface of PTAP2, and its mechanical integrity remains well preserved.


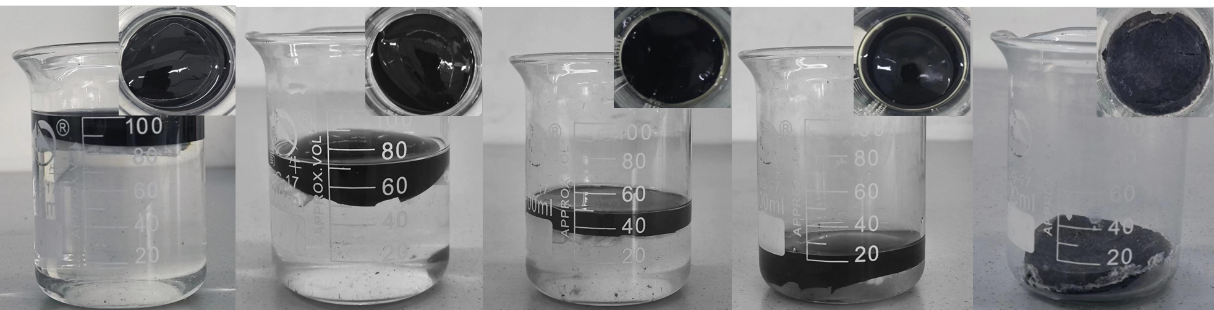


**Fig. S16** Side and front photographic views of PTAP2 for continuous seawater desalination until the solid salt is separated from water, and the origin salinity of seawater is 3.5 wt% brine

PTAP2 is subjected to long-term SID experiments in a 3.5 wt% brine (100 mL). As the initial brine continues to evaporate, solid salt particles first crystallize on the surface of the glass beaker. Until the brine is completely evaporated, no salt crystallization occurs on the surface of PTAP2.


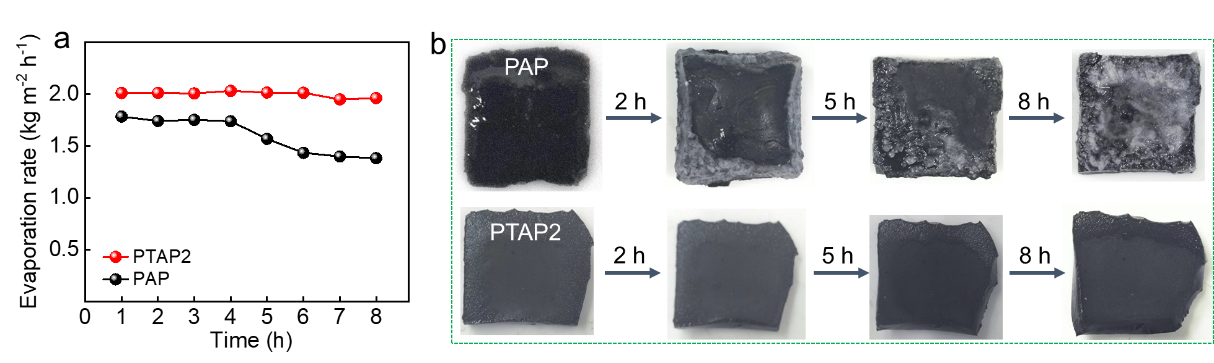


**Fig. S17** Long-term evaporation rates (a) and surface saltation (b) of PTAP2 and PAP in 20 wt% brine

Continuous water evaporation performance tests are conducted for PAP and PTAP2 in 20 wt% high-salinity brine for 8 h to observe the formation of salt crystallization on their surfaces. Even after 8 h of continuous operation in 20 wt% brine, PTAP2 exhibits no significant salt accumulation, and its evaporation rate remains stable. In contrast, under the same conditions, the evaporation interface of PAP is almost entirely covered with solid salt, leading to a 22.5% decrease in the water evaporation rate. In PTAP2, the zwitterionic PTMAO features closely positioned positively charged N⁺ and negatively charged O^−^. The O^−^ engages in strong hydrogen bonding with water molecules, enhancing their transport to the photothermal interface and thereby accelerating evaporation. Simultaneously, the N^+^ effectively repels salt ions, preserving the superhydrated shell and ensuring excellent nonfouling performance during continuous SID.

**
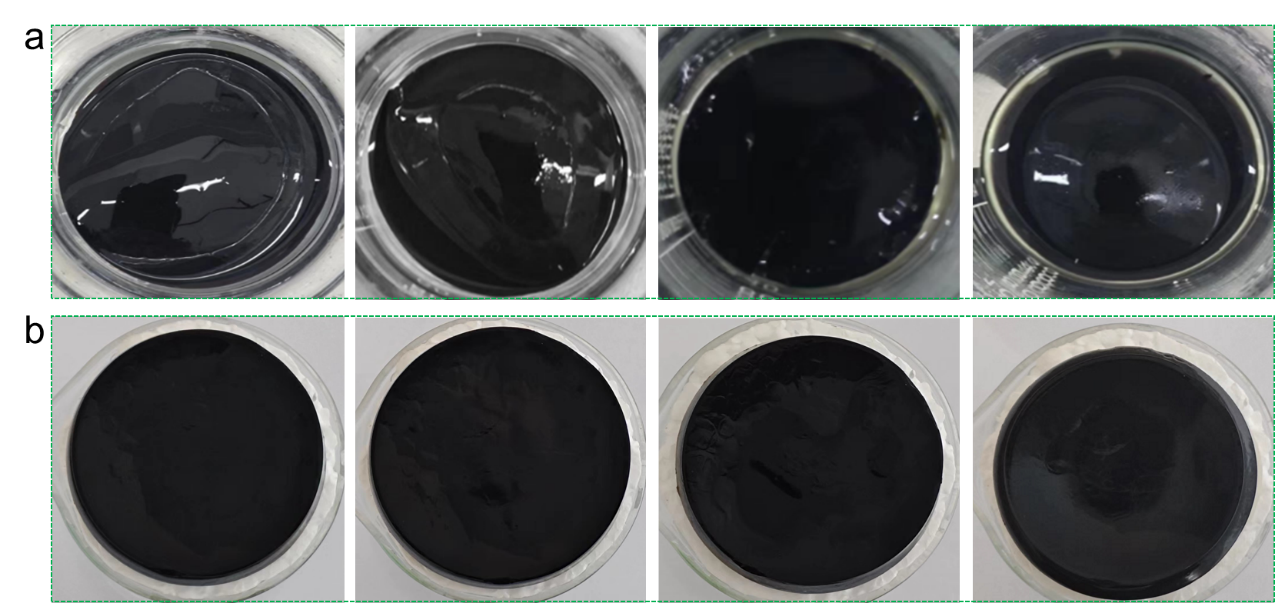
**

**Fig. S18** Photograph of PTAP2 during continuous operation in both (a) 3.5 wt% and (b) 10 wt% NaCl solutions, respectively

During the continuous operation in both 3.5 wt% and 10 wt% NaCl solutions, the morphology of PTAP2 was systematically monitored through photographic documentation before and after testing. No visible swelling, deformation, or structural collapse was observed over the testing period. In our experiments, a floating polystyrene foam substrate was employed to support PTAP2, ensuring stable floatation at the water-air interface throughout the testing period.

**
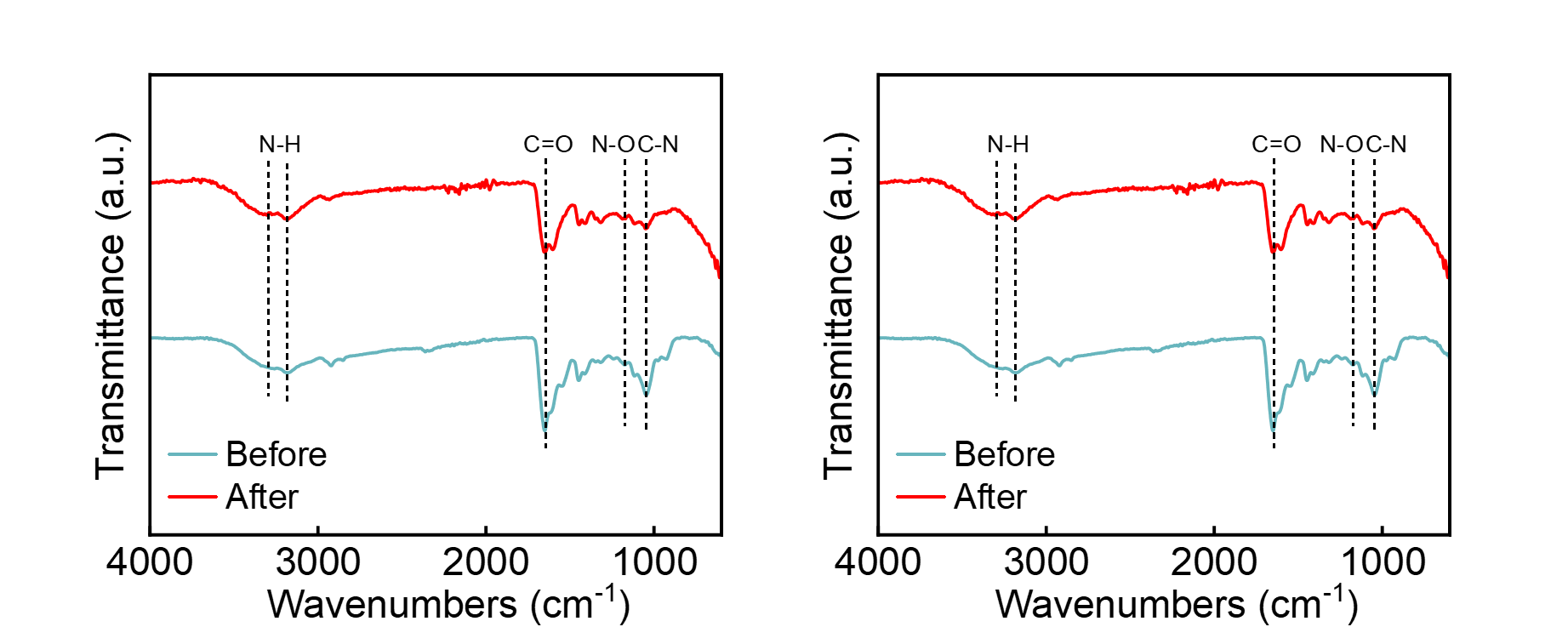
**

**Fig. S19** FTIR spectra of PTAP2 before and after continuous operation for 100 h in seawater

Notably, under 1 kW m^–2^, the surface temperature of PTAP2 in the SID system remains at ~37 °C. Such a low operational temperature ensures that the internal polymeric network of PTAP2 remains structurally intact during solar desalination. Furthermore, FTIR analysis was conducted to evaluate the chemical stability of PTAP2 after continuous operation for 100 h in seawater. The results reveal no observable shifts in the characteristic peaks of the major functional groups, confirming the PTAP2’s excellent chemical stability throughout the solar desalination process.

**Table S1** Salt-resistant performance summary of PTAP2 with previously reported solar evaporators

| Anti-salt solar evaporators | Evaporation rate  (kg m^−2^ h^−1^) | Durability  (h) | Refs. |
| --- | --- | --- | --- |
| rGO/PVA | 2.5 | 3.5 wt% NaCl for 96 h | **[S**6] |
| Spatially isolating salt crystallizer | 1.42 | 3.5 wt% NaCl for 600 h | [S7] |
| CNTs@SiO_2_ nanofibrous aerogels | 1.50 | 3.5 wt% NaCl for 1 h | [S8] |
| WHS | 1.27 | 10 wt % NaCl for 432 h | [S9] |
| Bionicgill evaporator | 2.11 | 10 wt% NaCl for 140 h | [S10] |
| Biomimetic 3D bridge-arch solar evaporator | 1.64 | 10 wt% NaCl for 200 h | [S11] |
| 3D open architecture | 1.44 | 10 wt% NaCl for 168 h | [S12] |
| PTAP2 | 2.15 | 3.5 wt% NaCl for 100 h | This work |
| PTAP2 | 2.23 | 10 wt% NaCl for 100 h | This work |

A summary of recently reported salt-resistant solar evaporators is provided for comparison. Notably, the designed PTAP2 hydrogel exhibits a clear advantage in evaporation rate under both low salinity (3.5 wt%) and high salinity (10 wt%) NaCl conditions compared with previously reported solar evaporators.

**Mechanism Exploration of Salt Resistance Using MD Simulations**


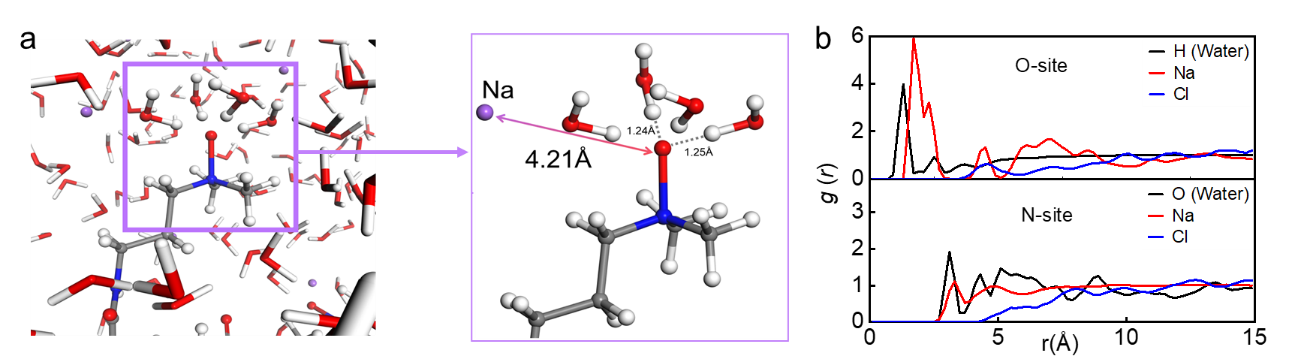


**Fig. S20** (**a**) MD snapshot illustrating the indirect interaction between a Na⁺ ion and the oxygen atom of TMAO. (**b**) Radial distribution function g(*r*) profiles for water hydrogen atoms, Na^+^, and Cl^−^ ions surrounding the oxygen (O-site) and nitrogen (N-site) of TMAO in a 3.5 wt% NaCl aqueous solution, based on MD simulations

In PTAP2, the zwitterionic PTMAO contains adjacent positively charged N^+^ and negatively charged O^−^ groups. The O^−^ group forms strong hydrogen bonds with water molecules, facilitating their migration to the photothermal interface and promoting rapid evaporation. Meanwhile, the N^+^ group effectively repels salt ions, preserving the integrity of the hydration shell and maintaining outstanding antifouling properties during continuous SID operation. Firstly, to study the mechanism accounting for the extraordinary strong hydration induced salt resistant of PTAP2, we performed molecular dynamics (MD) simulations of a TMAO small molecule in a saline solution. Unlike the exposed oxygen atom, the N^+^ in TMAO is shielded by three methyl groups and does not interact with surrounding water molecules. Therefore, the water molecules directly associated with the oxygen atom are primarily responsible for TMAO’s strong hydration. Approximately three hydrogen bonds are formed between water molecules and the oxygen atom of TMAO, contributing to the formation of a stable hydration shell. The persistent binding of several water molecules with the TMAO structure indicates the presence of highly robust interactions with water.

The radial distribution function g(*r*) displays a distinct peak between the oxygen atom of TMAO and the hydrogen atoms of surrounding water molecules, situated within 1.0–1.7 Å, confirming the presence of strong hydrogen bonding [S13]. Analysis of g(*r*) peak intensities around the O- and N-sites of TMAO indicates that Na^+^ cations associate more strongly with TMAO than Cl^−^ anions. Notably, Na^+^ shows a dominant peak near the O-site at 2.0–3.0 Å, suggesting a preference for direct coordination with the O^−^ group over indirect interactions via its hydration shell. In contrast, both Na^+^ and Cl^−^ exhibit only weak interactions near the N-site. Furthermore, there is no observable direct interaction between the quaternary N^+^ group and water molecules. The close spatial arrangement of the O^−^ and N^+^ groups in TMAO creates electrostatic repulsion that hinders strong Na^+^ binding to the O^−^ site. This combined spatial and electrostatic effect implies that the hydration shell of TMAO serves as a barrier to Na^+^ penetration, resulting in minimal alteration of its hydration structure upon NaCl addition.


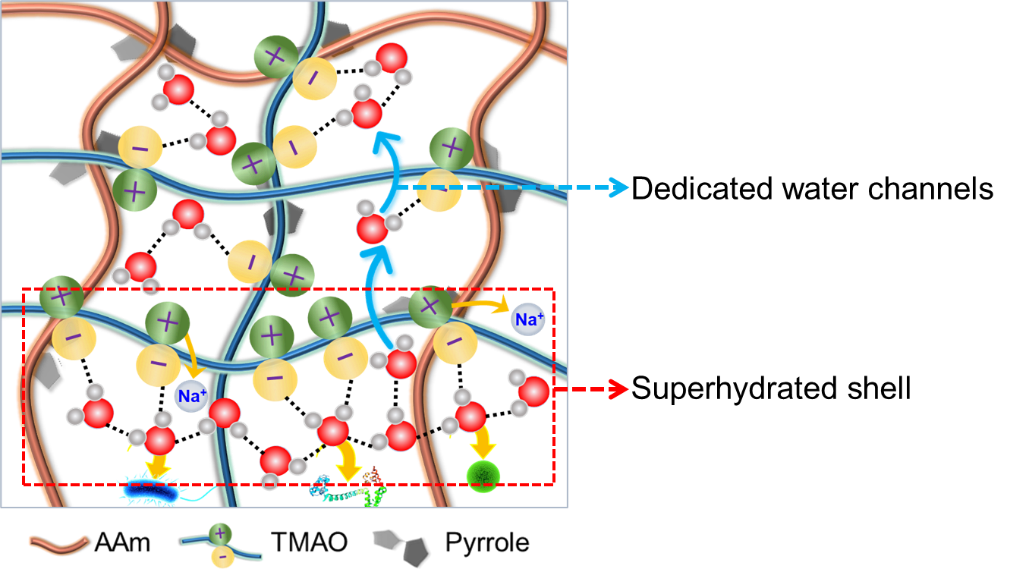


**Fig. S21** Schematic illustration of superhydrated PTAP with dedicated water channels for nonfouling SID

Benefiting from the directly connected positively charged N⁺ and negatively charged O^−^ of PTMAO, PTAP2 forms dedicated water channels that facilitate selective water molecule transport during evaporation. Meanwhile, the electrostatic repulsion from the positively charged N⁺ groups, combined with the strong hydration of the negatively charged O⁻ sites, effectively inhibits the ingress of salt ions and the attachment of foulants including proteins, bacteria, and algae. The excellent nonfouling performance of PTAP2 demonstrates its outstanding salt resistance and strengthens its stability and durability in long-term SID.

**MD Simulations Based on the LAMMPS**


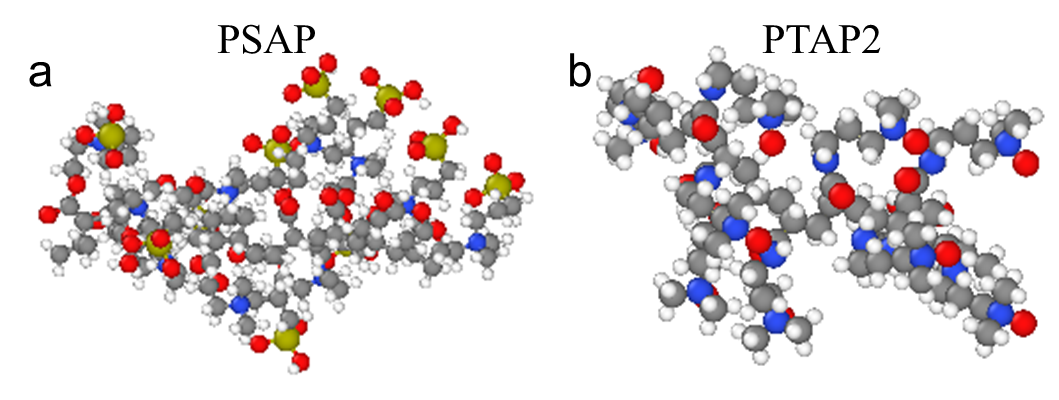


**Fig. S22** MD model construction of (**a**) PSAP and (**b**) PTAP2


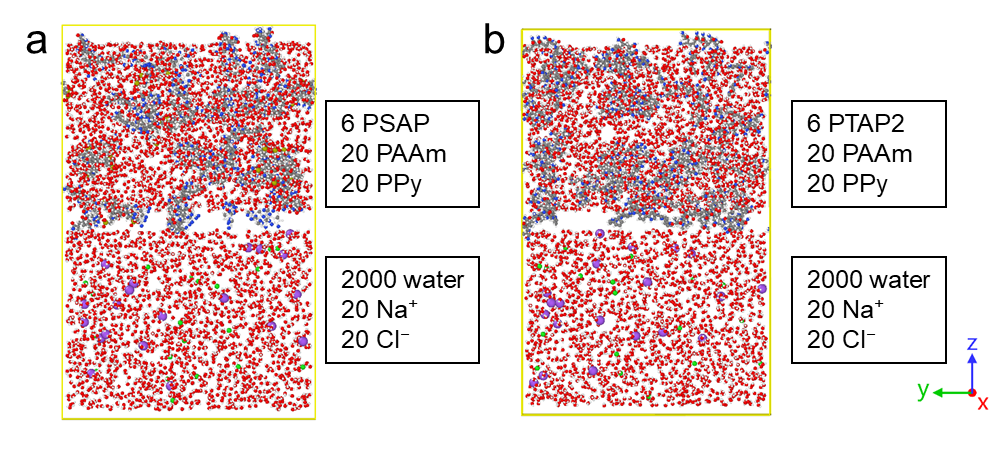


**Fig. S23** MD model construction of (**a**) 6 PSAP, 20 PAAm, 20 PPy, 2000 water, 20 Na^+^ 20 Cl^−^, and (**b**) 6 PTAP2, 20 PAAm, 20 PPy, 2000 water, 20 Na^+^ 20 Cl^−^

MD simulations were conducted using the Large-scale Atomic/Molecular Massively Parallel Simulator (LAMMPS) to explore the evaporation behavior of water molecules and the salt resistance properties of PSAP and PTAP2 systems [S14]. PTAP2 and PSAP were synthesized under comparable conditions to ensure similar crosslinking density and water content, except for the difference in zwitterionic monomer (PTMAO vs. PSBMA). Both hydrogels were prepared using the same total monomer concentration, crosslinker ratio (MBAA), and initiator content. Therefore, the observed differences in water transport and ion rejection are primarily attributed to molecular-level hydration effects of the zwitterionic units rather than bulk network architecture. In the process of MD model construction, the network morphologies of PTAP2 and PSAP are similar. Both the molecular weights of PTAP2 (molecular weight=1750) and PSAP (molecular weight=1710) are also similar. Six PTAP2 and 6 PSAP are added separately into the simulation box of PTAP2 and PSAP, respectively. And 20 PAAm, 20 PPy, and 2000 water molecules are also included. The lower part of the simulation box is salt solution, which contains 2000 water molecules, 20 Na^+^ and 20 Cl^−^. Thus, the network morphologies, water content, and crosslinking densities between PTAP2 and PSAP are similar. Since the molecular weight of PTAP2 (include 10 N^+^-O^−^) is smaller than PSAP (include 6 N^+^-SO_3_^−^), the hydration and salt resistant of PTAP2 is stronger than PSAP for the same mass dosing. The influences of polymeric architecture were not included in the current work, which can be investigated in the following work.


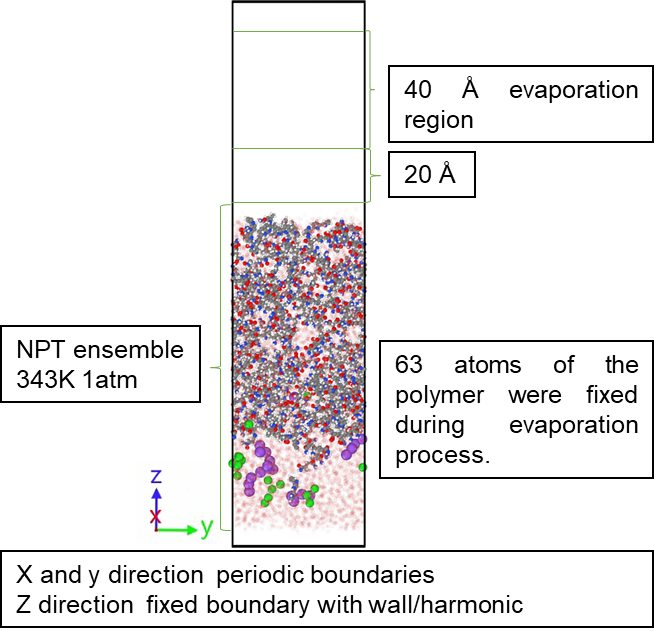


**Fig. S24** Detailed MD simulation parameters

The time step of MD simulation is set to be 1fs. The x and y directions use periodic boundaries, while the z direction uses fixed boundaries. Harmonic walls (wall/harmonic) are set at both the upper and lower boundaries to prevent atom loss. To maintain the positions of the PSAP and PTAP2 systems in the MD simulation, we fixed 63 atoms (PTAP2, PAAm, and PPy have approximately 6,300 atoms, with approximately 1% of atoms fixed). The remaining systems were simulated using the NPT ensemble, with Nose-Hoover temperature and pressure controllers and a damping coefficient of 1000. The evaporation process was simulated under conditions of controlled pressure in the x and y directions at T = 343 K and p = 1 atm (1 atm = 101.3 kPa), with the evaporation temperature maintained at 37 °C, below the boiling point of water, to accelerate the simulation. An evaporation region was defined 20–60 Å above the polymer liquid surface, and water molecules entering this region were deleted to quantify evaporation. The temperature was maintained at 37 ^o^C to enhance the evaporation rate while remaining below the boiling point of water. The simulation was conducted for 6 ns, and trajectory data were subsequently analyzed. The number of water molecules evaporated from the system can be determined by the change in the total number of particles in the MD trajectory file. The two systems are set up identically to facilitate comparison of steady-state evaporation rate and ion migration behavior.

The primary distinction between the two systems lies in the molecular species of sulfobetaine methacrylate (SBMA) and TMAO. A rectangular simulation box with dimensions of 100 × 100 × 150 Å was constructed, wherein the upper half was randomly populated with six TMAO molecules, twenty PAAm molecules, twenty PPy molecules, and 2000 water molecules. The lower half was filled with 2000 water molecules, twenty chloride ions, and twenty sodium ions. Polymer parameters were assigned based on the GAFF2 force field and xtb-resp charge calculation method, while water molecules were modeled using the TIP4P/ε framework. Force field parameters and atomic charges were generated using the AutoFF software to ensure a dynamically equilibrated model. Following structural optimization, dynamic relaxation was sequentially performed under the canonical (NVT) and isothermal-isobaric (NPT) ensembles. During the NVT phase, a Nose-Hoover thermostat was applied at 363 K with a temperature damping coefficient of 1000 for 200 ps, with fixed boundaries and reflective walls imposed along the z-axis. Subsequently, under the NPT ensemble, the system was relaxed using a Nose-Hoover thermostat coupled with a Berendsen barostat, maintaining T = 343 K and p = 1 atm (1 atm = 101.3 kPa) with pressure control in the x and y directions for 200 ps. To further stabilize the structure, multilayer graphene sheets were placed at the top and bottom of the system, applying a compressive force equivalent to 0.02 kcal mol^−1^, followed by removal of the graphene and additional relaxation under the NPT ensemble (T = 343 K, p = 1 atm) with lateral pressure control. Upon completion of optimization and relaxation, the system energy stabilized, and the simulated density closely matched experimental observations.


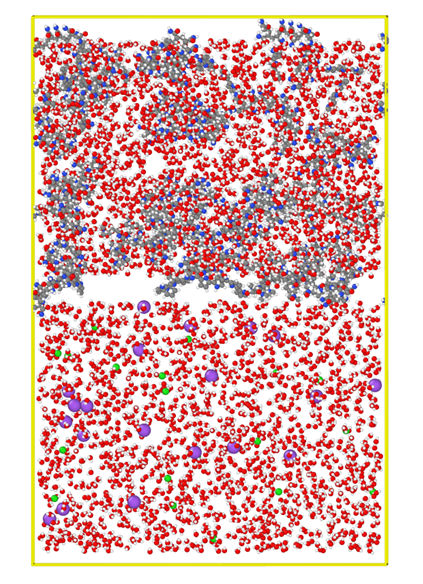


**Fig. S25** Schematic illustration of the initial simulation model


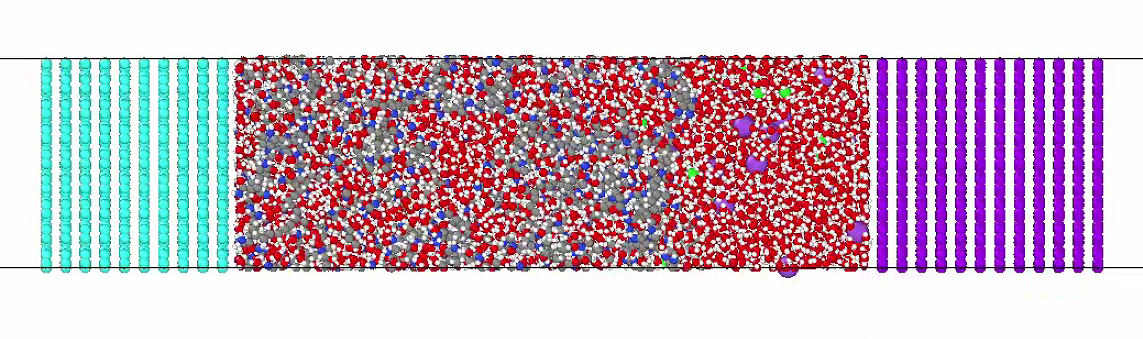


**Fig. S26** Schematic illustration of the initial simulation model compressed along the Z-direction

**Evaporation Conditions**


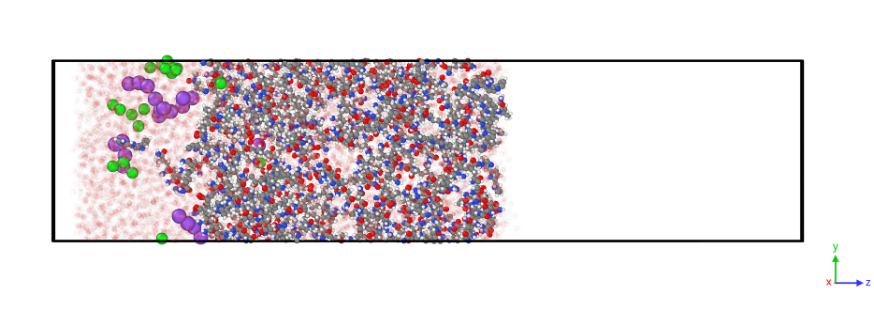


**Fig. S27** Diagram of the initial stage of a simulated evaporation system

During the evaporation simulation, the periodic boundary condition in the Z-direction was removed, and reflective walls were applied to the top and bottom boundaries of the simulation box to prevent atom loss. A 100-Å vacuum slab is set above the water interface to collect evaporated water molecules. An evaporation region was defined 20–60 Å above the polymer liquid surface, and water molecules entering this region were deleted to quantify evaporation. The temperature was maintained at 37 ^o^C to enhance the evaporation rate while remaining below the boiling point of water. The simulation was conducted for 6 ns, and trajectory data were subsequently analyzed. Interaction energies between water molecules, salt ions, and the polymer were calculated using the “compute group/group” command. Trajectories were visualized with the open visualization tool (OVITO), and the data were further analyzed with visual molecular dynamics (VMD) to investigate changes in hydrogen bonding during evaporation.


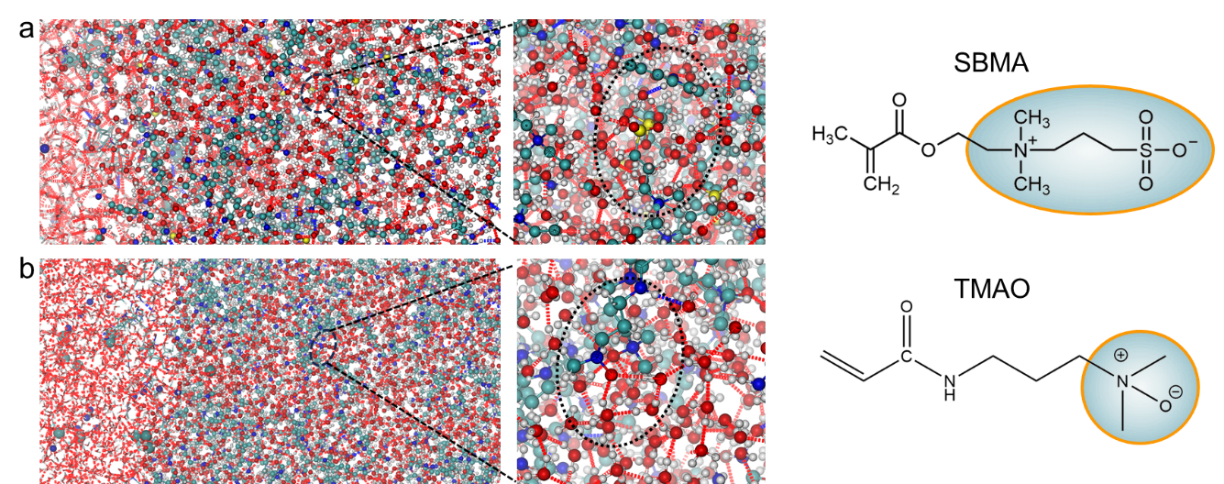


**Fig. S28** Schematic diagram of simulated water molecule transport in (**a**) PSAP and (**b**) PTAP2 in 3.5 wt% NaCl solution. The enlarged image shows the strong hydrogen bonding of PSBMA in PSAP and PTMAO in PTAP2 with surrounding water molecules, respectively.

The MD simulation results show that in PSAP, the relatively large distance and pronounced steric hindrance between the cationic functional group (–N⁺(CH_3_)_3_) and the anionic functional group (–SO_3_⁻) of PSBMA result in the formation of approximately two hydrogen bonds with surrounding water molecules. In contrast, the directly connected N⁺ and O⁻ groups within PTMAO in PTAP2 enable the formation of about three hydrogen bonds with nearby water molecules, thereby promoting the development of a stable hydration shell. Meanwhile, the total number of hydrogen bonds in PTAP2 are greater than that of PSAP, further confirming its stronger hydration ability for highly salt-resistant SID.


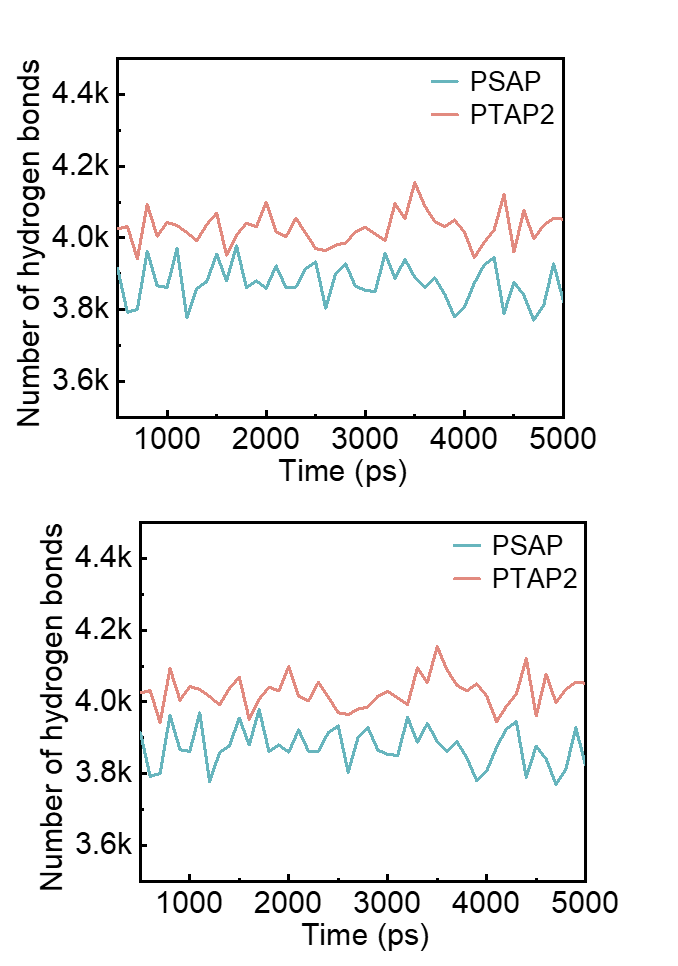


**Fig. S29** The number of hydrogen bonds in PSAP and PTAP2 calculated based on the final 5000 ps of the simulation trajectory


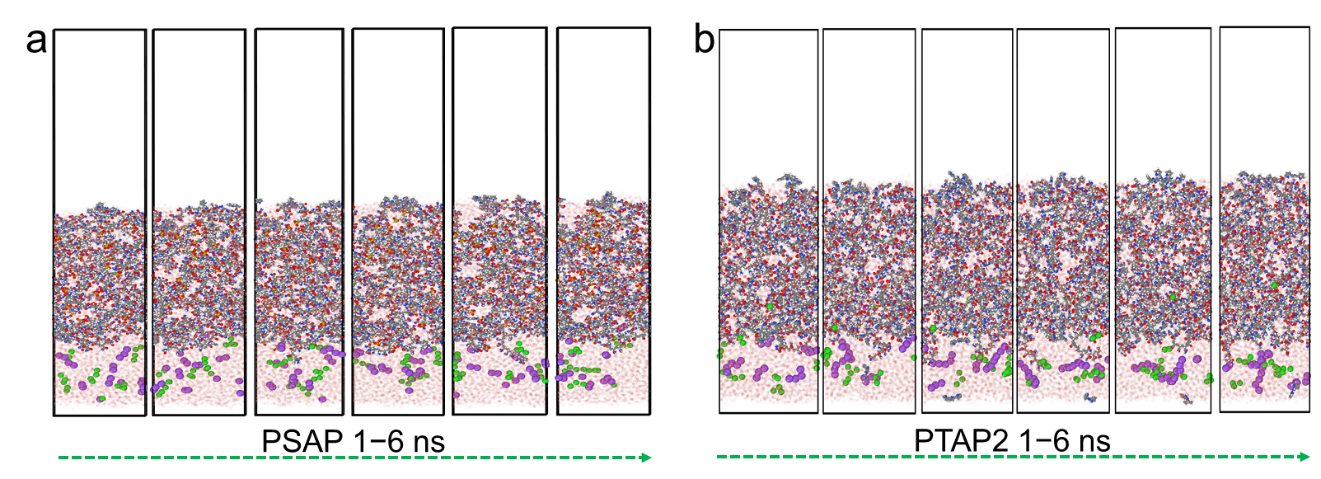


**Fig. S30** Simulated snapshots of the salt ions states during evaporation in (a) PSAP and (b) PTAP2

Subsequently, based on the established evaporation models of PSAP and PTAP2 in a 3.5 wt% NaCl solution, we simulated the transport of salt ions and the evaporation of water molecules. The corresponding behaviors were further analyzed by calculating the free energy profiles of salt ions and water molecules within the PSAP and PTAP2 systems. The potential of mean force (PMF), representing the free energy along the evaporation direction (z-direction), was calculated as *G*(*z*) = −*k*_B_*T* ln(*ρ*(*z*)/*ρ*_ref_), where *k*_B_ is the Boltzmann constant, *T* is the temperature, and *ρ*(*z*) is the water density (with a unit of nm^−3^) at a given position z. The reference position, *z*_ref_, was chosen at the point of minimum free energy. The PMF profile of salt ions (Na^+^ and Cl^−^) were obtained using the umbrella sampling method. A harmonic biasing potential, *k*/2·(*z*−*z*_i_)^2^, was applied to ions, where *z* is the *z*-coordinate of the salt ion, *z*_i_ is the equilibrium position of the spring, and *k* is the force constant (2.00 kcal mol^−1^ Å^−2^ in these calculations). The equilibrium positions vary from *z*_i_ = 0 to 58 with an increment of 2 Å, resulting in 30 sampling windows (*z* = 18 Å corresponds to the center of the PTAP2). For each window, equilibrium MD simulations were performed for 15 ns. The final 20 ns of data from each window were analyzed using the weighted histogram analysis method (WHAM) to reconstruct the PMF profiles.

As water molecules evaporate, no substantial salt ions are transported to the evaporation interface in PSBMA and PTAP2 systems. At the liquid/solid interface, the PMF for PTAP2-Na⁺ and PTAP2-Cl⁻ is notably higher than that for PSAP-Na⁺ and PSAP-Cl⁻, suggesting a greater transmembrane energy barrier and enhanced salt resistance in PTAP2. At the solid/gas interface, the PMF value of PSAP-H_2_O is higher than that of PTAP2-H_2_O, suggesting that water molecules evaporate more readily and in greater quantities in the PTAP2-H_2_O system. Moreover, at the evaporation interface, water molecules in the PSAP system require a higher energy barrier to escape (−4 to −2.5 kJ mol^−1^), whereas the corresponding energy barrier in the PTAP2 system is notably lower (−2.5 to −1 kJ mol^−1^). Consistently, MD simulations reveal that the number of evaporated water molecules is substantially greater in the PTAP2 system compared to the PSAP system. These findings demonstrate that the incorporation of PTMAO, featuring directly linked N^+^ and O⁻ groups, facilitates the formation of molecular-scale water channels through robust hydrogen bonding with water molecules in the PTAP2 system. This unique architecture not only accelerates water evaporation but also endows the PTAP2 with superior antifouling performance, thereby enhancing its potential for SID applications in complex marine environments.


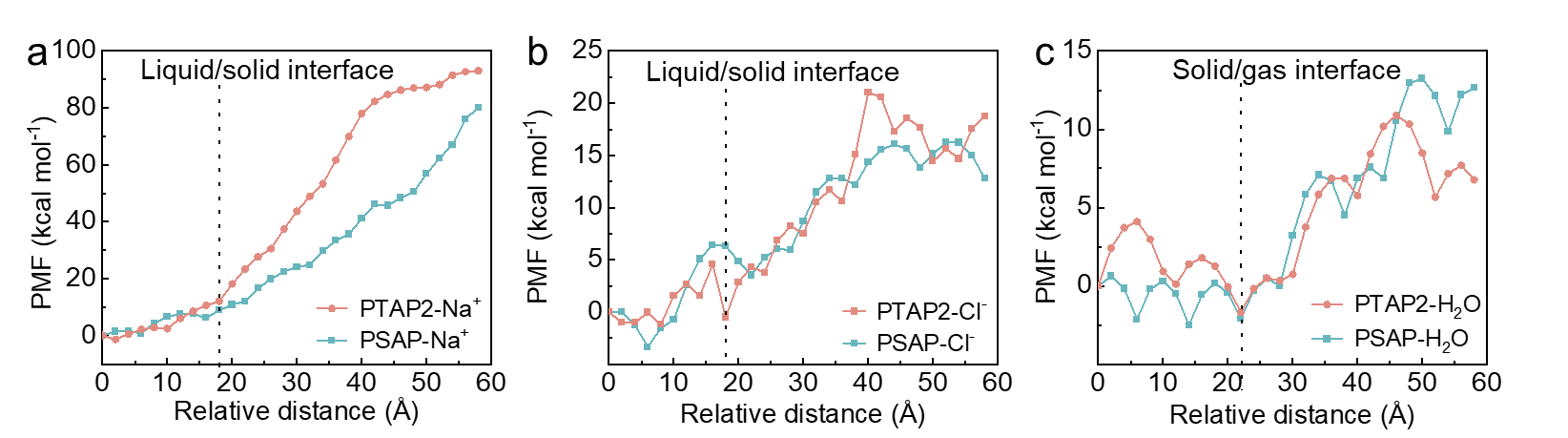


**Fig. S31** PMF curves of (**a**) PSAP-Na^+^ and PTAP2-Na^+^, (**b**) PSAP-Cl^−^ and PTAP2-Cl^−^ at the liquid/solid interface, and (**c**) PSAP-H_2_O and PTAP2-H_2_O at the solid/gas interface along the direction of evaporation, respectively


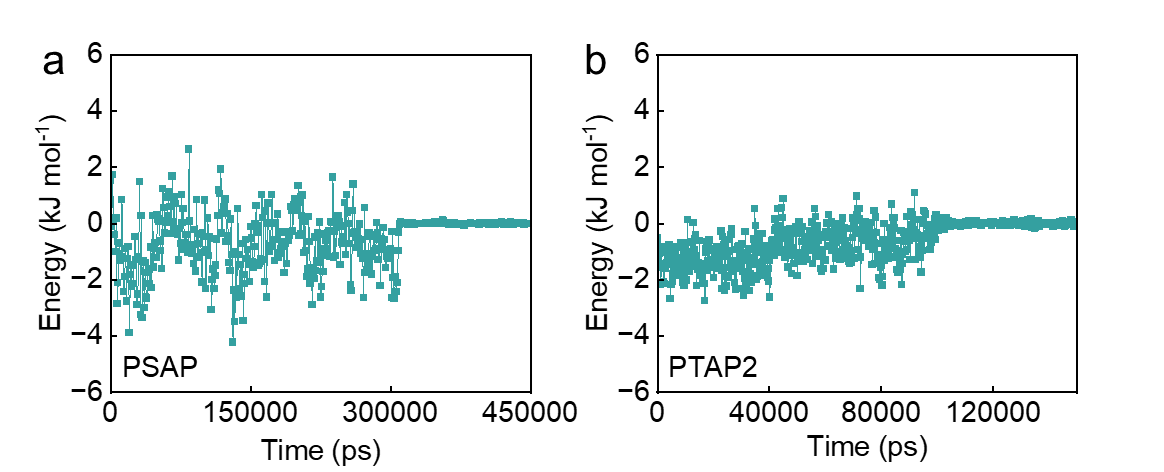


**Fig. S32** At the solid/gas interface, the energy required for water vapor escape from (**a**) PSAP and (**b**) PTAP2, respectively


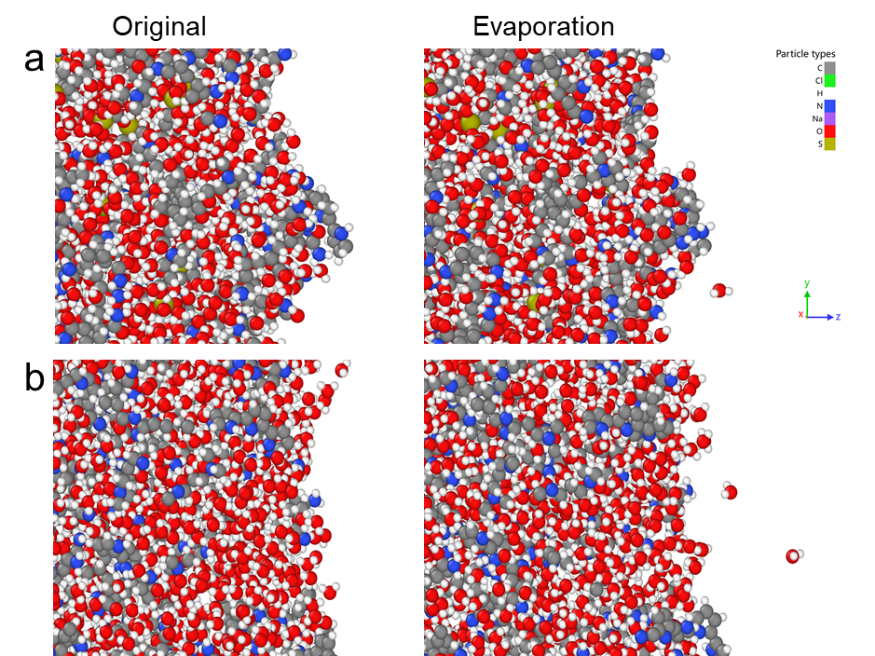


**Fig. S33** Simulated snapshots of original state and water evaporation in (**a**) PSAP and (**b**) PTAP2 systems


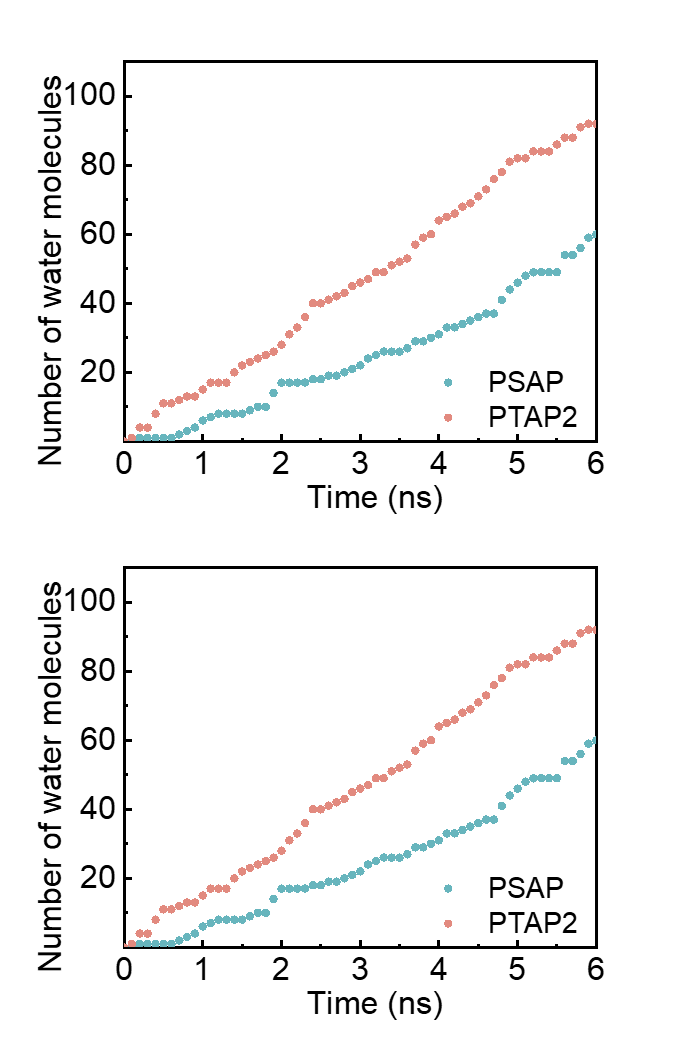


**Fig. S34** The quantity of water molecules that evaporated across the solid/gas interface in PSAP and SBMA in a 3.5 wt% NaCl solution based on the last 6 ns of MD simulation data


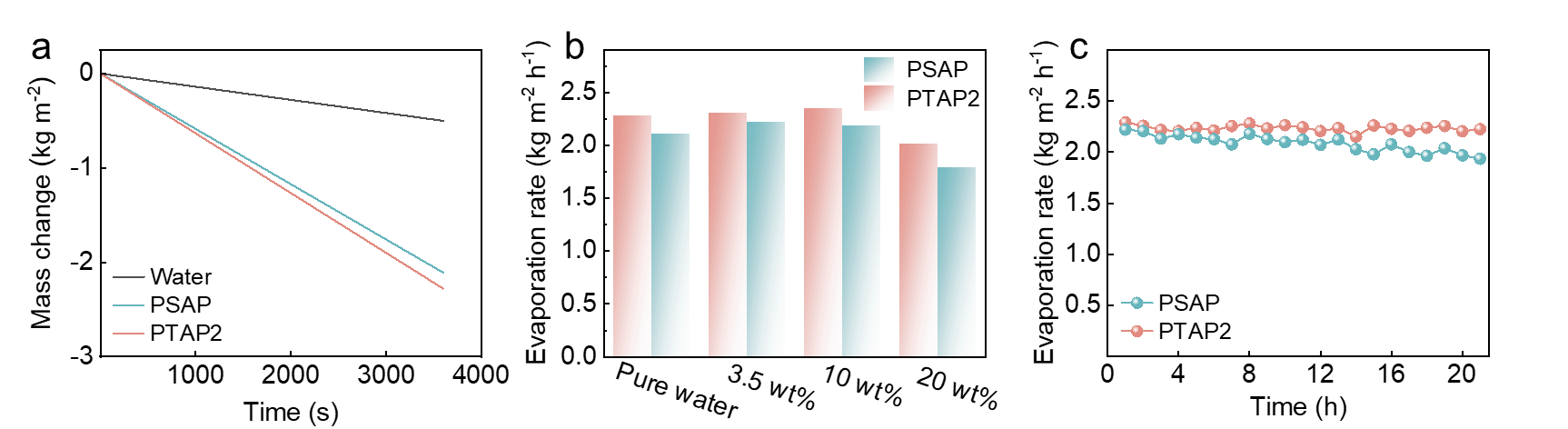


**Fig. S35** (**a**) Mass change of pure water, PSAP, and PTAP2 versus time. (**b**) Evaporation rate of PSAP and PTAP2 in pure water and different brines. (**c**) Long-term evaporation rate of PSAP and PTAP2 in 3.5 wt% NaCl solution under 1 kW m^–2^


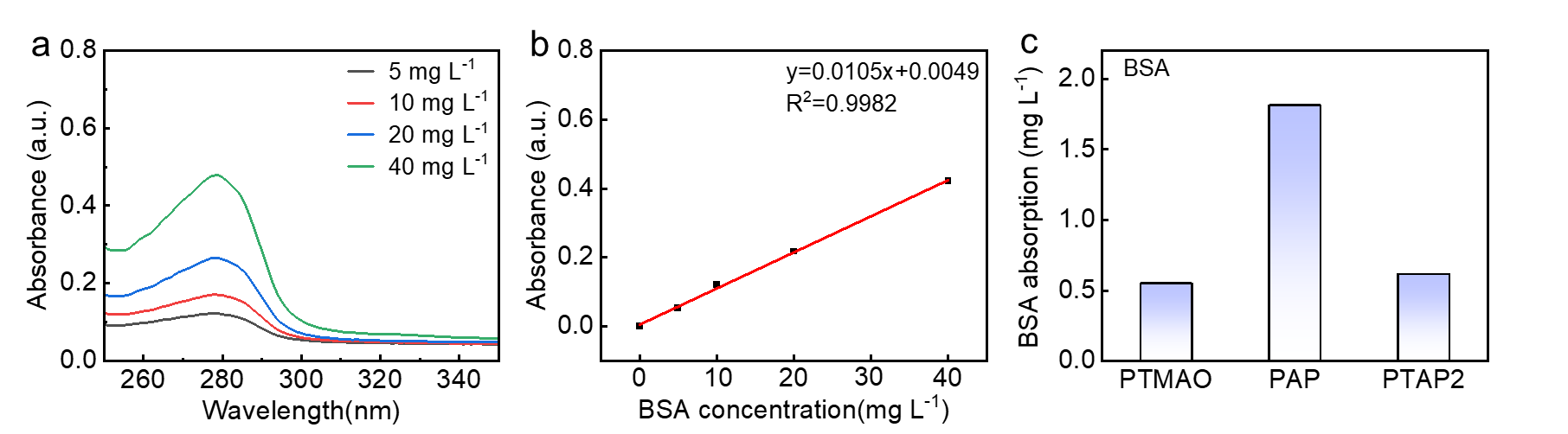


**Fig. S36** (**a**) UV-Vis absorption spectra of different concentrations of BSA solutions. (**b**) absorbance-concentration standard curve of BSA solutions

The absorbance of BSA solutions at different concentrations is measured at λ = 280 nm to establish a standard curve using UV-vis spectroscopy. After the PTAP2 surface reaches stable adsorption, PBS buffer is used to gently rinse off loosely bound BSA from the PTAP2 surface, followed by rinsing with deionized water to further dilute any remaining BSA. The absorbance of BSA per unit area on the PTAP2 surface is then measured to evaluate the antifouling performance of the hydrogel against protein adhesion. In the anti-protein adhesion assay, the amount of BSA adsorbed on the PTAP2 surface reaches only 0.61 mg cm^−2^, comparable to that on PTMAO (0.55 mg cm^−2^) but significantly lower than that on PAP (1.81 mg cm^−2^). This enhanced antifouling performance is primarily attributed to the electrostatic neutrality of the zwitterionic PTMAO, which minimizes interactions with negatively charged BSA. Moreover, the strong hydration capacity of PTMAO promotes the formation of a dense and continuous superhydrated layer on the PTAP2 surface, effectively serving as a physical barrier against BSA adsorption.


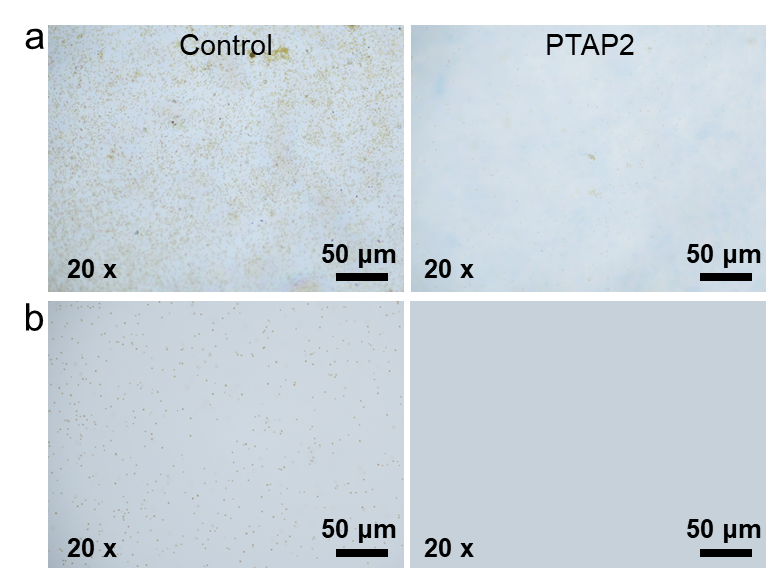


**Fig. S37** Optical microscopy images of (**a**) Diatoms and (**b**) Chrysophytes adhesion on the surfaces of blank control and PTAP2 after coincubation

We additionally examined the performance of PTAP2 against diatoms and chrysophytes. Notably, no visible fouling is observed on the surface of PTAP2 after prolonged exposure to solutions containing either Diatoms or Chrysophytes, demonstrating the excellent durability and nonfouling capability of PTAP2 in complex marine environments.

**Outdoor Water Collection Using Actual Seawater**


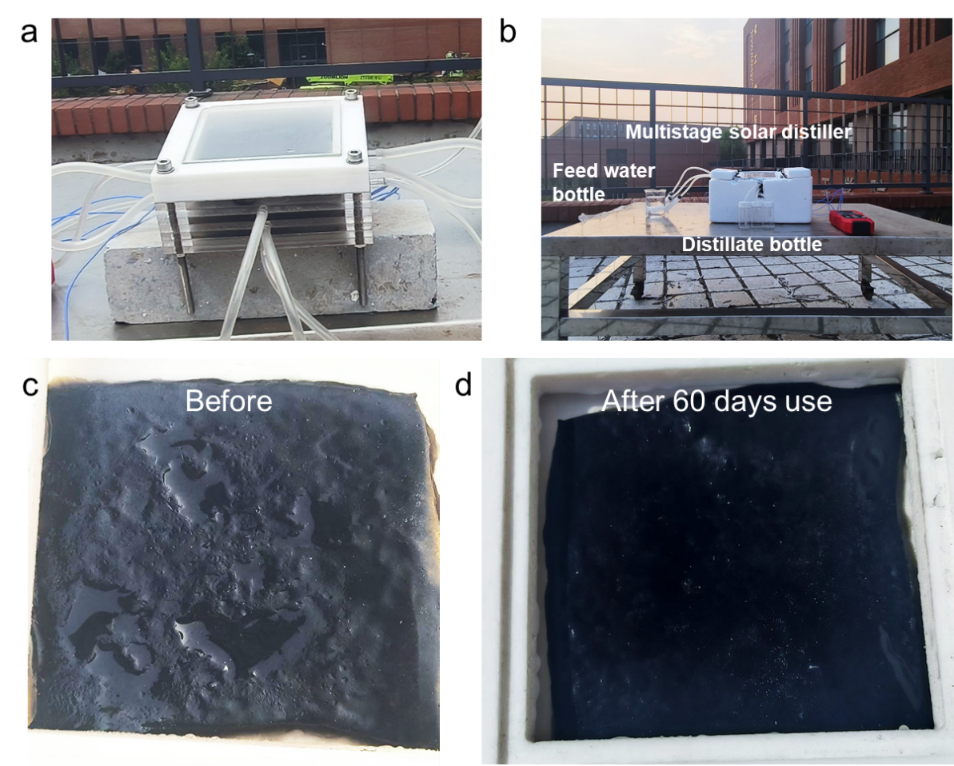


**Fig. S38** (**a, b**) Photographs of the designed multistage solar distiller. (**c, d**) Photographs of PTAP2 before and after 60 days use

We designed a three-stage solar distillation unit for long-term clean water collection in outdoor environments. The first-stage distillation unit comprises a transparent glass, a PTFE frame, the photothermal material PTAP2, a water inlet layer, a PTFE hydrophobic membrane (which selectively allows water vapor to pass), a drainage layer, and a condensing aluminum plate. The second and third stages each consist of an inlet layer, a hydrophobic membrane, a drainage layer, and a condensing aluminum plate. All stages are connected by screw holes located at the four corners of the inlet and drainage layers. PTAP2 serves as the photothermal material, absorbing solar energy and transferring heat downward. The hydrophobic membrane prevents liquid leakage from the inlet layer while enabling water vapor transmission. To mitigate performance losses caused by vapor condensation on light-transmitting surfaces, the transparent glass and condensation plates are coated with a hydrophobic layer. Additionally, the drainage layers are directly connected to the hydrophobically treated condensation plates, ensuring rapid vapor condensation and efficient collection of liquid water. The entire multistage solar distiller unit is wrapped in insulating foam to minimize thermal losses.

During the test period, PTAP2 was sealed within the chamber of solar distiller, which was constructed from chemically inert PTFE to ensure long-term stability under continuous solar irradiation. As a result, no surface cleaning or replacement of the PTAP2 was required. While minor dust occasionally settled on the transparent glass cover, natural rainfall effectively removed it, eliminating the need for manual cleaning.


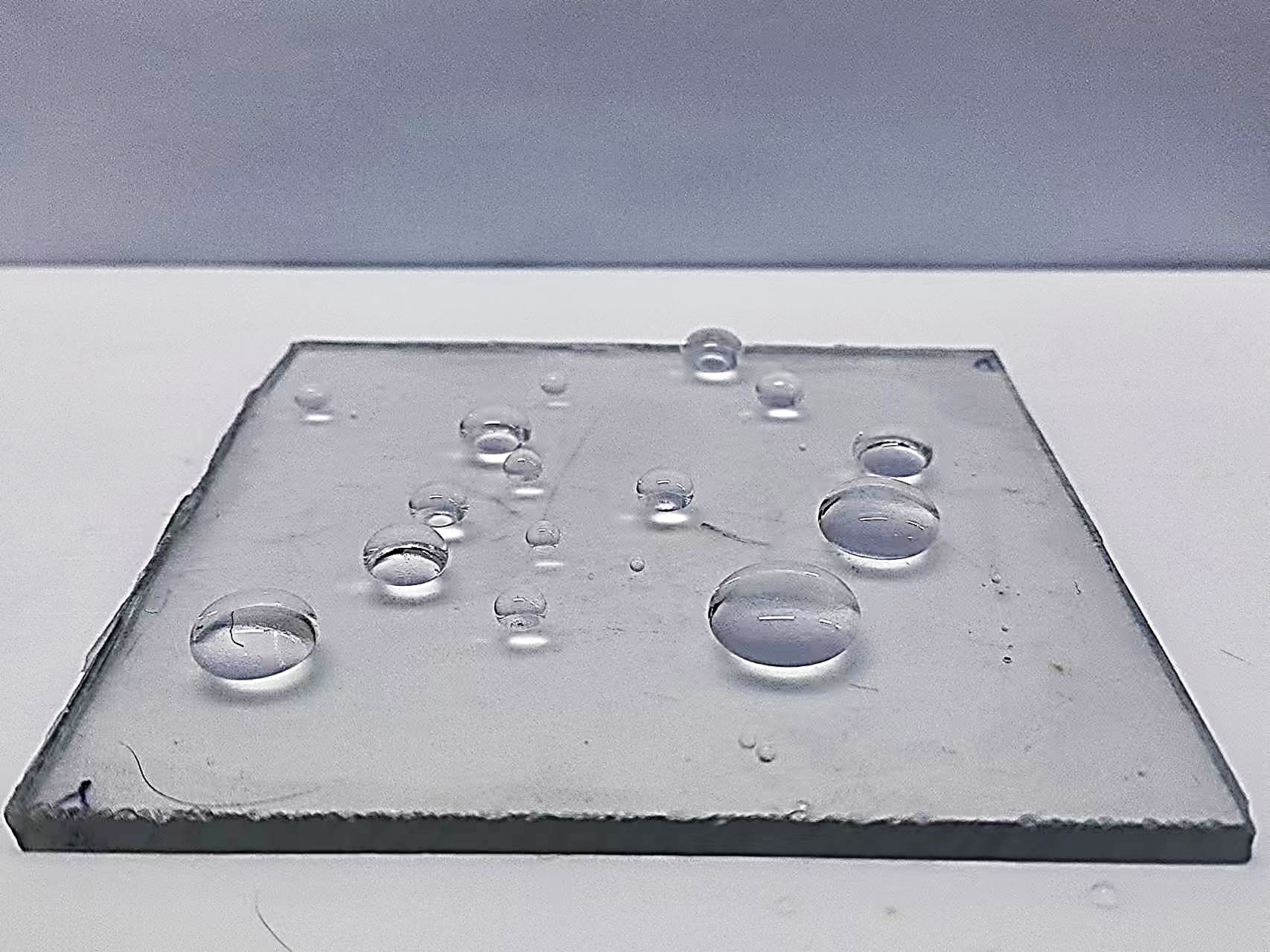


**Fig. S39** Photograph of transparent glass surface after hydrophobic modification

To prevent performance degradation caused by vapor condensation, such as reduced light absorption and hindered liquid water collection, the transparent glass and aluminum condensation plates were modified with a superhydrophobic coating developed within our research group. The coating suspension primarily consists of SiO_2_ nanoparticles, 1H,1H,2H,2H-Perfluorodecyltrimethoxysilane (PFS, 98%), and Ethanol glue (the main component is acrylic synthetic resin) [S15]. Superhydrophobic surfaces were fabricated by directly spray-coating the suspension onto the target substrates using an airbrush. This method is facile, scalable, and capable of producing large-area superhydrophobic surfaces with minimal processing steps.

**Supplementary References**

1. B. Li, P. Jain, J. Ma, J.K. Smith, Z. Yuan et al., Trimethylamine N-oxide-derived zwitterionic polymers: a new class of ultralow fouling bioinspired materials. Sci. Adv. **5**(6), eaaw9562 (2019). <https://doi.org/10.1126/sciadv.aaw9562>
2. X. Li, G. Ni, T. Cooper, N. Xu, J. Li et al., Measuring conversion efficiency of solar vapor generation. Joule **3**(8), 1798–1803 (2019). <https://doi.org/10.1016/j.joule.2019.06.009>
3. Z. Yu, X. Li, X. Li, B. Zheng, D. Li et al., Nacre-inspired metal-organic framework coatings reinforced by multiscale hierarchical cross-linking for integrated antifouling and anti-microbial corrosion. Adv. Funct. Mater. **33**(47), 2305995 (2023). <https://doi.org/10.1002/adfm.202305995>
4. P. Zhang, H. Wang, Z. Xia, S. Xing, J. Li et al., Hydrogen-bond-repairing solar evaporator with reconstructed large-width channels for durable solarizing seawater. Nano Lett. **24**(37), 11615–11623 (2024). <https://doi.org/10.1021/acs.nanolett.4c03179>
5. C. Lei, W. Guan, Y. Guo, W. Shi, Y. Wang et al., Polyzwitterionic hydrogels for highly efficient high salinity solar desalination. Angew. Chem. Int. Ed. **61**(36), e202208487 (2022). <https://doi.org/10.1002/anie.202208487>
6. X. Zhou, F. Zhao, Y. Guo, Y. Zhang, G. Yu, A hydrogel-based antifouling solar evaporator for highly efficient water desalination. Energy Environ. Sci. **11**(8), 1985–1992 (2018). <https://doi.org/10.1039/C8EE00567B>
7. Y. Xia, Q. Hou, H. Jubaer, Y. Li, Y. Kang et al., Spatially isolating salt crystallisation from water evaporation for continuous solar steam generation and salt harvesting. Energy Environ. Sci. **12**(6), 1840–1847 (2019). <https://doi.org/10.1039/C9EE00692C>
8. X. Dong, L. Cao, Y. Si, B. Ding, H. Deng, Cellular structured CNTs@SiO_2_ nanofibrous aerogels with vertically aligned vessels for salt-resistant solar desalination. Adv. Mater. **32**(34), 1908269 (2020). <https://doi.org/10.1002/adma.201908269>
9. N. Xu, J. Li, Y. Wang, C. Fang, X. Li et al., A water lily-inspired hierarchical design for stable and efficient solar evaporation of high-salinity brine. Sci. Adv. **5**(7), eaaw7013 (2019). <https://doi.org/10.1126/sciadv.aaw7013>
10. L. Zhang, Y. Zhang, M. Zou, C. Yu, C. Li et al., A bionic-gill 3D hydrogel evaporator with multidirectional crossflow salt mitigation and aquaculture applications. Adv. Funct. Mater. **33**(24), 2300318 (2023). <https://doi.org/10.1002/adfm.202300318>
11. M. Zou, Y. Zhang, Z. Cai, C. Li, Z. Sun et al., 3D printing a biomimetic bridge-arch solar evaporator for eliminating salt accumulation with desalination and agricultural applications. Adv. Mater. **33**(34), e2102443 (2021). <https://doi.org/10.1002/adma.202102443>
12. K. Yang, T. Pan, S. Dang, Q. Gan, Y. Han, Three-dimensional open architecture enabling salt-rejection solar evaporators with boosted water production efficiency. Nat. Commun. **13**(1), 6653 (2022). <https://doi.org/10.1038/s41467-022-34528-7>
13. H. Huang, C. Zhang, R. Crisci, T. Lu, H.-C. Hung et al., Strong surface hydration and salt resistant mechanism of a new nonfouling zwitterionic polymer based on protein stabilizer TMAO. J. Am. Chem. Soc. **143**(40), 16786–16795 (2021). <https://doi.org/10.1021/jacs.1c08280>
14. Q. Lyu, D.-Y. Kang, S. Hu, L.-C. Lin, Exploiting interior surface functionalization in reverse osmosis desalination membranes to mitigate permeability–selectivity trade-off: Molecular simulations of nanotube-based membranes. Desalination **491**, 114537 (2020). <https://doi.org/10.1016/j.desal.2020.114537>
15. X. Qin, L. Kong, J. Wang, M. Liu, S. Lu et al., Double-layer with electroactive and superhydrophobicity based on polymer nanotubes to improve robustness and anti-corrosion performances. Chem. Eng. J. **488**, 150928 (2024). <https://doi.org/10.1016/j.cej.2024.150928>
